# Staphylococcal exoribonuclease YhaM destabilizes ribosomes by targeting the mRNA of a hibernation factor

**DOI:** 10.1093/nar/gkae596

**Published:** 2024-07-09

**Authors:** Anna Lipońska, Hyun Lee, Mee-Ngan F Yap

**Affiliations:** Department of Microbiology-Immunology, Northwestern University Feinberg School of Medicine, 320 E Superior St, Chicago, IL 60611, USA; Department of Pharmaceutical Sciences, College of Pharmacy and Biophysics Core in Research Resources Center, University of Illinois at Chicago (UIC), 1100 S Ashland Ave, Chicago, IL 60607, USA; Department of Microbiology-Immunology, Northwestern University Feinberg School of Medicine, 320 E Superior St, Chicago, IL 60611, USA

## Abstract

The hibernation-promoting factor (Hpf) in *Staphylococcus aureus* binds to 70S ribosomes and induces the formation of the 100S complex (70S dimer), leading to translational avoidance and occlusion of ribosomes from RNase R-mediated degradation. Here, we show that the 3′-5′ exoribonuclease YhaM plays a previously unrecognized role in modulating ribosome stability. Unlike RNase R, which directly degrades the 16S rRNA of ribosomes in *S. aureus* cells lacking Hpf, YhaM destabilizes ribosomes by indirectly degrading the 3′-*hpf* mRNA that carries an intrinsic terminator. YhaM adopts an active hexameric assembly and robustly cleaves ssRNA in a manganese-dependent manner. *In vivo*, YhaM appears to be a low-processive enzyme, trimming the *hpf* mRNA by only 1 nucleotide. Deletion of *yhaM* delays cell growth. These findings substantiate the physiological significance of this cryptic enzyme and the protective role of Hpf in ribosome integrity, providing a mechanistic understanding of bacterial ribosome turnover.

## Graphical Abstract

**Graphical Abstract:**
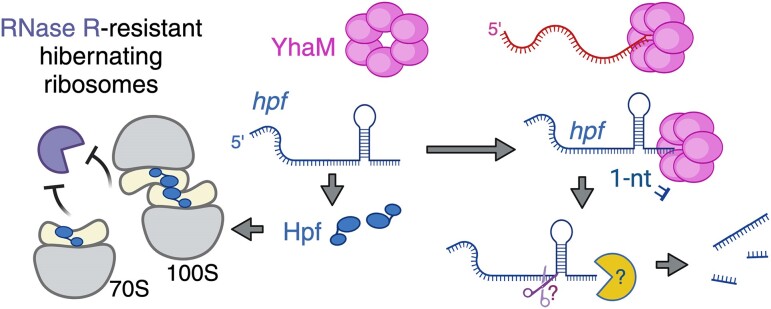

## Introduction

Ribosome hibernation is a conserved survival mechanism used by bacteria and eukaryotes to either sequester mature 70S or 80S ribosomes in a translationally incompetent state (1–7) or to suppress active translation (8). Depending on the classes of hibernation factors, bacterial hibernating ribosomes can exist as a translationally inactive 70S ribosome or a dimer of 70S ribosomes (100S complex) (8–17). Most bacteria, including the human opportunistic pathogen *Staphylococcus aureus*, use a long form hibernation-promoting factor (Hpf) to induce the 30S-to-30S conjoining of 70S ribosomes through the dimerization of Hpf C-terminal domains (CTD) on the opposing copies of 70S monomers, whereas the Hpf N-terminal domain (NTD) occupies the decoding sites of the 30S subunits, blocking the entry of the mRNA and tRNA to exclude translation (12,14). Despite the variation in hibernation factors and the distinct dimeric architecture of bacterial 100S complexes (Supplementary Table S1) (7), the loss of hibernation factors often leads to defects in long-term viability and regrowth, reduced host infections, and sensitivity to antibiotics and a wide range of stressors (3,15,18–26). These defects were initially thought to be linked to dysregulated translation. However, global translation in *S. aureus* is only modestly affected in the absence of Hpf (21), implying that translational exclusion does not play a pivotal role in cell survival. Recent studies have confirmed that the primary role of Hpf and other bacterial hibernation factors is to maintain ribosome integrity because a short-lived knockout either undergoes rapid ribosome degradation or carries partially damaged 70S ribosomes (13,18,19,21,27–30). In *S. aureus*, the 100S ribosomes are resistant to nucleolytic cleavage by the 3′-5′ exoribonuclease RNase R (31), whereas *Escherichia coli* hibernating ribosomes are protected from the concerted action of RNase R and an endonuclease YbeY at a few functionally important 16S rRNA sites that are distinct from those found in *S. aureus* (Supplementary Table S1) (32). Unlike most bacteria, *S. aureus* Hpf is expressed throughout its life cycle that is primarily driven by the CodY transcription factor (21,33–36). Ribosome hibernation can be reversed by disassembly factors or in a passive manner to allow reentry of ribosomes into the translational cycle during stationary phase when nutrient is limited (3,20,37,38). In *S. aureus*, the levels of 100S complexes are partially reduced by the sequestration of Hpf by a glutamate dehydrogenase-like YwlG (39). *S. aureus* lacking Hpf is impaired in murine colonization by three orders of magnitude (36). These results reinforce the importance of ribosome hibernation in preserving and priming ribosomes for translational reactivation.

We previously showed that inactivation of RNase R (among 13 other tested RNases) in the Δ*hpf* mutant does not fully restore the ribosome contents to wild-type (WT) levels, suggesting the existence of other unidentified ribosome degradation factors. In this study, we identify a 3′-5′ exoribonuclease, YhaM, in ribosome turnover and describe the biochemical features and substrate preference of YhaM that have previously been underappreciated. We show that deletion of *yhaM* reduces cell growth and that YhaM promotes ribosome decay by degrading the *hpf* mRNA upon trimming of its 3′-end. These findings reveal the physiological significance of a poorly characterized RNase and demonstrate an alternative pathway of ribosome turnover by curtailing a ribosome hibernation factor.

## Materials and methods

### Strains, plasmids, chemicals and growth conditions

*S. aureus* USA300 JE2 is a community-associated methicillin-resistant *Staphylococcus aureus* (CA-MRSA, GenBank CP000255). The ribonuclease mutants carry a *bursa aurealis* transposon insertion were acquired from BEI Resources and are listed in the Supplementary Table S2. The *S. aureus* mutants were constructed as previously described (31), all mutant strains were verified by Sanger sequencing and Western blots.

To overexpress His_6_-tag YhaM protein, primers P1440/1441 were used to amplified ∼940-bp *yhaM* using JE2 genomic DNA as a template and cloned into an IPTG-inducible pMCSG7 via a ligation independent approach (40). To construct a complementation plasmid carrying *yhaM* under the control of its own native promotor, primer pairs 1684/1684 (5′-UTR), 1686/1687 (*yhaM*) and 1688/1689 (pLI50) were used to perform cloning with a Gibson Assembly kit (New England Biolabs). To introduce HD-domain mutations, primer pairs 1692/1693 (H192A), 1694/1695 (D193A) and 1823/1824 (H192A/D193A) were used. Primers and RNA oligonucleotides were purchased from IDT DNA and are listed in Supplementary Table S3.

*S. aureus* were grown at 37°C in tryptic soy broth (TSB, Difco) at 5:1 tube- or flask-to-medium ratio with a 1:100 dilution of an overnight seed culture. *E. coli* were grown in LB (Difco). When necessary, erythromycin, chloramphenicol, kanamycin, ampicillin and IPTG were used at 5 μg/ml, 10 μg/ml, 75 μg/ml, 100 μg/ml and 0.5 mM, respectively. All key chemicals, reagents and instrumentation are listed in Supplementary Tables S4–S7.

### Ribosome sedimentation profiles

Crude ribosomes were isolated from *S. aureus* by cryo-milling methods in Buffer A [20 mM HEPES (pH 7.5), 14 mM Mg(OAc)_2_, 100 mM KCl, 0.5 mM PMSF, 1 mM DTT] (21). Five absorbance units (Abs_260_) of ribosomes were layered on a 5–30% sucrose gradient that was prepared on a BioComp Gradient Master. The samples were centrifuged at 210 000×g at 4°C SW41 rotor in a Beckman Coulter Optima XPN-100 ultracentrifuge for 3 hr. Fractionation was performed using Brandel fraction system equipped with a UA-6 UV detector. To quantitate the abundance of total ribosomes particles relative to the singe *Δhpf* mutant, the boundaries of ribosomal peaks were manually selected from the trough between peaks. The total area under a peak was calculated by ImageJ and divided to obtain the ratio. When immunoblotting was needed, ∼200 μl per fraction were collected and subjected to final 10% trichloroacetic acid precipitation. The pellets were washed with cold acetone once, suspended in 50 mM Tris-base containing Laemmli sample buffer and resolved by 4–20% TGX SDS-PAGE (BioRad).

### Construction of YhaM phylogenetic tree

The clusters of orthologous genes (COGs) (41) database was used to obtain YhaM sequences. The non-redundant sequences were removed manually. A total of 258 *yhaM* genes from 234 distinct bacteria or archaea were extracted for multiple-sequence alignment using MUSCLE algorithm in Jalview 2.11.2.6 software (42) using default parameters. Phylogenetic analysis were built by MEGA11 software (43) using Maximum Likelihood method and the JTT matrix based model, with partial deletion of positions containing gaps and missing data.

### Antibodies and western blots

Cell pellets from 10 ml of TSB *S. aureus* cultures were homogenized with Lysing Matrix B (MP Biomedicals) in 20 mM Tris (pH 7.5) on a FastPrep-24 homogenizer (MP Biomedicals) for 1 cycle of 40 s at speed of 4M/s. Clarified lysates were recovered by spinning at 20 817×g at room temperature for 2 min to remove cell debris. A total of 0.75 Abs280 units of cell lysate were analyzed on 4–20% TGX SDS-PAGE gels (BioRad) and the proteins were transferred to a nitrocellulose membrane using a Trans-Blot Turbo system (BioRad). The membrane was stained with Ponceau red (Amresco #K793-500ml) to ensure equal loading, followed by immunoblotting using anti-YhaM (1/2000 dilutions), anti-S11 (1/2000–1/4000 dilutions) (21), anti-Hpf (1/5000–1/8000 dilutions) (33). To generate anti-YhaM, two peptides corresponding to 74–95 and 288–313 residues of the *S. aureus* YhaM were custom synthesized: [Cys-NYRGNKQMKVNQIRLATTEDQLK; Cys-KTDKGQFTDKIFGLENRRFYNPESLD] and used for immunization in New Zealand white rabbits (Pacific Immunology). HRP-conjugated anti-IgG secondary antibody (1/15000 dilutions) was from Cytiva (#NA9120) and SuperSignal™ West Dura chemiluminescence substrate (Thermo Scientific #34075) was used for signal detection. Alexa Fluor Plus 800 anti-Rabbit IgG antibody (1/10 000) (Invitrogen #A32735) was used when YhaM quantification was needed. Images were acquired using iBright FL1500 system (ThermoFisher). Gel analysis was performed using ImageJ software followed by statistical analysis using paired *t*-test in GraphPad Prism Software v9.

### Overexpression and purification of recombinant YhaM

The overexpression and purification of the His-tagged recombinant proteins using Ni-NTA affinity chromatography have been described in detail previously (21,37). Selected fractions of purified His-tagged YhaM were loaded on a Amicon Ultra Centrifugal filter unit (MWCO-10 Millipore) to concentrate the proteins in buffer B [40 mM HEPES (pH 7.5), 0.5 M KCl, 10% glycerol]. Proteins were analyzed on 4–20% TGX SDS-PAGE (BioRad) and stained with GelCode™. LC–MS/MS mass spectrometry was performed by the Northwestern University Proteomics Core to identify the truncated YhaM fragment. Scaffold Software was used to validate MS/MS based peptide and protein identification. For size-exclusion chromatography, a total of 60 μM YhaM protein was loaded on HiPrep^TM^ 16/60 Sephacryl S-100 HR column (Cytiva) on an ÄKTA Start chromatography system (Cytiva) and equilibrated with buffer B [40 mM HEPES (pH 7.5), 0.5M KCl, 10% glycerol]. To estimate the size of YhaM peaks a Gel Filtration Standard (BioRad #151-1901) was used.

### Total RNA purification

Ten milliliters of TSB cultures were centrifuged and washed twice with 1 × volume of cold killing buffer [20 mM Tris–HCl (pH 7.5), 5 mM MgCl_2_, 20 mM NaN_3_]. Cells were resuspended in 0.5 ml T_10_E_1_ buffer and disrupted on a Fastprep-24 homogenizer (MP Biomedicals) for 1 cycle of 60 s at speed of 4M/sec using Lysing Matrix B beads (MP Biomedicals). The samples were extracted 3 times with acid phenol/chloroform (pH 4.5) and once with chloroform/isoamyl-alcohol (24:1). The final aqueous phase was precipitated with 1 × volume of isopropanol 1/10 volume of 3M NaOAc (pH 5.2, Alfa Aesar) and final RNA pellets were washed once with 70% ethanol. RNA integrity was analyzed on a 0.8–1% TAE denaturing agarose gel and stained with ethidium bromide. RiboRuler High Range RNA ladder (ThermoFisher #SM1821) was used to estimate RNA size.

### Determination of steady-state mRNA by Northern Blot

A modified nonradioactive detection method was used for Northern Blots (44). Three micrograms of RNA were treated with Turbo DNase I and RNA integrity was confirmed by analysis on a 1% TAE denaturing agarose gel and stained with ethidium bromide. RiboRuler High Range RNA ladder (ThermoFisher #SM1821) was used to estimate RNA size. Samples were preheated for 5 min at 95°C. Gel was transferred on a positively charged nylon membrane (Ambion #AM10104) for 2 h with 5 × SSC buffer, followed by crosslinking with UV Stratalinker™ 2400 at a default autocrosslink of 1200 μJ/cm^2^. Membrane was stained with methylene blue. Hybridization was performed using 3′-DIG synthetic oligonucleotide P1943 (Supplementary Table S3) using QuikHyb (Agilent) hybridization buffer at 42°C for minimum 12 hr. Membranes were washed twice with 4 × SSC at 42°C. Further steps were following DIG Northern Starter Kit (Roche) protocol. Images were acquired using iBright FL1500 system (ThermoFisher) and analyzed using ImageJ software followed by statistical analysis using Paired t test in GraphPad Prism Software v9.

### Mapping rRNA cleavages sites by primer extension

Ribosomal complexes were fractionated by sucrose density ultracentrifugation as described above from cultures growth for 12 h at 37°C. The 70S peaks were subjected to acidic phenol-chloroform extractions, and rRNAs were precipitated by isopropanol. Two hundred and fifty nanograms of total rRNA was used for primer extension as described previously (31) using the 5′-end fluorescently labeled antisense oligos (Supplementary Table S3). DNA sequencing ladders were generated using a USB Thermo SEQ kit (Affymetrix) with 16S rDNA as a template. The reverse transcribed products were heat denatured and resolved on 10% TBE-Urea polyacrylamide sequencing gels and then scanned on a Typhoon 5 Imager (Cytiva). Secondary structure of *S. aureus* 16S rRNA was obtained from RNAcentral database (45).

### Measurement of mRNA half-lives

*S. aureus* cells were grown in TSB at 37ºC until OD_600_= 1.5. Four milliliters of cultures were taken as untreated control. Thirty milliliters of culture were transferred to a new flask and treated with a final concentration of 400 μg/ml rifampicin. At 1, 2, 3, 4, 5 and 8 min post-rifampicin treatment, 4 ml cultures were mixed with 5 × volumes of ice-cold acetone-ethanol (1:1 ratio) to stop the reaction. Cell pellets were harvested by centrifugation at 4ºC at 3220 × g for 15 min, supernatant was aspirated, and residual liquid was fully removed after additional short spin. A 0.5 ml ice-cold acetone-ethanol (1:1 ratio) was layered on top of the cell pellets and samples were stored at –80°C until RNA purification. Total RNA was extracted using the modified hot phenol-SDS method (46–48). Cells were suspended in 1 ml protoplast buffer [50 mM Tris–HCl pH 7.5; 25% (w/v) sucrose; 0.25 mM EDTA, fresh 50 μg/ml lysostaphin (AMBI, #LSPN-50)] and incubated on ice for 15 min. After 20 817×g centrifugation at 4°C for 5 min, pellet was resuspended in 1 ml buffer T_10_E_1_ [10 mM Tris–HCl (pH 7.5); 1 mM EDTA] and added to 0.5 ml preheated lysis buffer [200 mM NaCl; 2% (w/v) SDS; 16 mM EDTA] and incubated for 5 min at 95°C. Samples were extracted 3 times with acid phenol/chloroform (pH 4.5) and once with chloroform/isoamyl-alcohol (24:1). The final aqueous phase was precipitated with 1 × volume of isopropanol 1/10 volume of 3M NaOAc (pH 5.2, Alfa Aesar) and final RNA pellets were washed once with 70% ethanol. Northern blots were performed as described above except that ULTRAhyb (Invitrogen AM8670) was used in place of the discontinued QuikHyb (Agilent). After hybridization, membrane was wash twice (5 min each) with 2 × SSC, twice (15 min) with [0.2 × SSC, 0.1% SDS] at 42ºC. To ensure equal RNA transfer, nylon membrane was either stained with methylene blue or hybridized with the 3′-DIG labeled 23S rRNA probe (oligo P1996, Supplementary Table S3). ImageJ software was used for analyzing *hpf* mRNA signal intensity. The data were normalized by dividing the T_0_ (estimated as 1 min post-rifampicin exposure). Log value was plotted on the *y*-axis versus time on the *x*-axis. Excel was used to add linear trendline function, in which *x* = *k*_decay_. The resulting value was used for equation: *t*_$\frac{1}{2}$_=log(2)/*k*_decay_.

### *In vitro* degradation of synthetic RNA, rRNA and ribosomal complexes

Reactions with 5′-(6-FAM) labeled RNA were carried out with 2.5 μM dsRNA or 1 μM ssRNA (Supplementary Table S3) in a total volume of 10 μl containing [50 mM Na-Tricine (pH 8.0), 100 mM KCl, 1 mM MnSO_4_ [(or 1 mM of CaCl_2_; MgCl_2_, MnCl_2_, NiCl_2_, CoSO_4_, CuSO_4_, FeSO_4_, MgSO_4_, ZnSO_4_)] and 200 nM of purified YhaM. dsRNA substrates were prepared by annealing RNA1 and RNA2 or RNA6 in Buffer D [300 mM KCl; 30 mM Tris–HCl (pH 7.5); 1 mM MgCl_2_] for 2 min at 95°C and slowly cooled down to 25°C (at 1°C/25 s). Control reactions were performed in reaction buffer without YhaM or with heat inactivated YhaM (at 95°C for 10 min). To prepare the single-nucleotide RNA marker, a 2.5 μM 6-FAM-RNA was hydrolyzed at 90°C for 5 min in alkaline buffer containing 0.5 M Na_2_CO_3_ and 10 mM EDTA. To prepare the guanine-specific RNA marker, the same RNA was digested with RNase T1 (Roche) in the presence of sodium citrate buffer for 10 min at 55°C. The same reactions were performed for rRNA degradation experiments except that a total of 2 μg of RNA were used per reaction with 0, 0.2, 1, 5 and 30 μM YhaM. For ribosome degradation, 1 pmol of ribosome was incubated with and without YhaM at a ribosome-to-protein molar ratio of 0, 1 and 5. The rRNA- and ribosome-containing reactions were analyzed on a 1% TAE agarose gel and stained with ethidium bromide.

### Electrophoretic mobility shift assay (EMSA)

Reaction with 15 nM 5′-(6-FAM) labeled RNA_a was incubated with YhaM at an increasing amount of YhaM (0, 1, 5, 7.5, 10, 12.5, 15, 17.5, 20, 25 and 30 μM) in a total volume of 20 μl containing [50 mM Na-Tricine (pH 8.0), 100 mM KCl, 1 mM MnSO_4_]. Control reactions were performed in reaction buffer without YhaM or with the heat inactivated YhaM (at 95°C for 10 min). Reactions were incubated at 37°C for 20 min and immediately loaded on a 20% TBE-native gel, in the presence of 10% glycerol loading solution. Fluorescence signals were visualized on an iBright™ FL1500 system (ThermoFisher).

### Circular RACE mapping of 3′- and 5′-termini

Circular RACE was used as described previously (49) with a few minor modifications. Briefly, total RNA was passed through a Qiagen RNeasy column for additional cleanup followed by Turbo™ DNase treatment. RNA was decapped with RppH (New England Biolabs; M0356S) for 30 min at 37°C. RNA circularization was performed with T4 RNA Ligase I (New England Biolabs M0204L) for 2 h at 25°C followed by 2 h at 37°C. Reaction was stopped by 95°C heating for 2 min. Reverse transcription was performed using SuperScriptTM III Reverse Transcriptase Kit (Invitrogen) and P1858 primer for 5 min at 25°C, 90 min at 50°C, 45 min at 55°C. The sample was inactivated by incubation at 70°C for 15 min and by RNase H (New England Biolabs, M0297L) treatment at 37°C for 20 min. cDNA fragment was PCR amplified twice using nested primer sets P1859/P1860 and P1856/P1857. The site of circulation reveals both 3′ and 5′ ends of the transcripts. PCR amplified product was then purified from 1% agarose gel with Gel/PCR DNA Fragments Extraction Kit (IBI Scientific) and subjected to Sanger DNA sequencing with P1856 or P1857. Sequencing results were analyzed using FinchTV software (version 1.4.0 Geospiza). All primer sequences are listed in (Supplementary Table S3).

### Mass photometry

Molecular weights and distribution of four samples (YhaM, HD, H and D correspond to YhaM^WT^, YhaM^H192A/D193A^, YhaM^H192A^ and YhaM^D193A^) were monitored with a TwoMP™ Mass photometer (Refeyn Ltd, Oxford, UK). The setup of the TwoMP™ mass photometer involved mounting a clean glass coverslip (Refeyn) onto it, followed by the careful placement of a 6-well Gasket (Refeyn) on top of the coverslip to complete the preparation process. To generate a calibration curve, the measurements of two standard proteins, β-amylase (BAM) with monomer, dimer, and tetramer forms (56, 112 and 224 kDa respectively), as well as thyroglobulin (TG) with dimer form (669 kDa), were obtained. A well in the gasket was filled with 14 μl of PBS, pH 7.4, and 6 μl of either BAM or TG was added and quickly mixed well followed by data collection for 60 s using the Acquire MP software. The measurements were made at a final concentration of 10 nM for both BAM and TG. The contrast-to-mass calibration curve had *R*^2^ = 0.99 and a mass error of 5.0%. Four samples (YhaM, HD, H and D) were initially diluted to 100 nM in six different buffers, NoMe (50 mM Na-Tricine, pH 8.0, 100 mM KCl), Mn^2+^ (50 mM Na-Tricine, pH 8.0, 100 mM KCl, 1 mM MnSO_4_), Mg^2+^(50 mM Na-Tricine, pH 8.0, 100 mM KCl, 1 mM MgSO_4_), and Co^2+^ (50 mM Na-Tricine, pH 8.0, 100 mM KCl, 1 mM CoSO_4_), and allowed to incubate while the instrument was calibrated with two standard proteins. Samples were monitored at a final concentration of 30 nM in wells of the same gasket and glass slide immediately after calibration in a same manner. Mass photometry movies were analyzed using DiscoverMP software (Refeyn). MP experiments were performed in a minimum of two biological replicates.

### *In vivo* glutaraldehyde crosslinking

Crosslinking experiments were adapted from published protocol (50). *S. aureus* cells were grown until OD_600_= 0.8. Pellet was resuspended in 40 mM HEPES (pH 7.5) and incubated with 2 mg/ml lysostaphin for 30 min at 37°C. Crosslinking was performed in the presence of Halt™ protease Inhibitor cocktail (Thermo Scientific). After addition of 1 mM of glutaraldehyde (Sigma, G5882) cells were incubated for 1 and 2 h at room temperature. At the endpoints, glutaraldehyde was quenched with 100 mM of Tris–HCl (pH 8.0) for 15 min at room temperature. Cells were homogenized with Lysing Matrix B (MP Biomedicals) on a FastPrep-24 homogenizer (MP Biomedicals) for 1 cycle of 40 s at speed of 4M/s. Clarified lysates were recovered by spinning at 20 817×g at room temperature for 2 min to remove cell debris. Total protein concentration was estimated with QuickStart Bradford Protein Assay (BioRad) and 75 μg of total proteins were analyzed on 4–12% Bis–Tris NuPAGE minigels (Invitrogen). Proteins were transferred to a PDVF membrane in buffer [25 mM Tris, 192 mM glycine, 0.1% SDS, 20% ethanol] using a Mini Trans-Blot Cell system (BioRad) at 4°C for 18–20 hr at 20V. Western blot was performed as described above.

## Results

### Deletion of *yhaM* (formerly *cbf1*) rescues the ribosome pools in a *Δhpf* knockout

**Figure 1.**
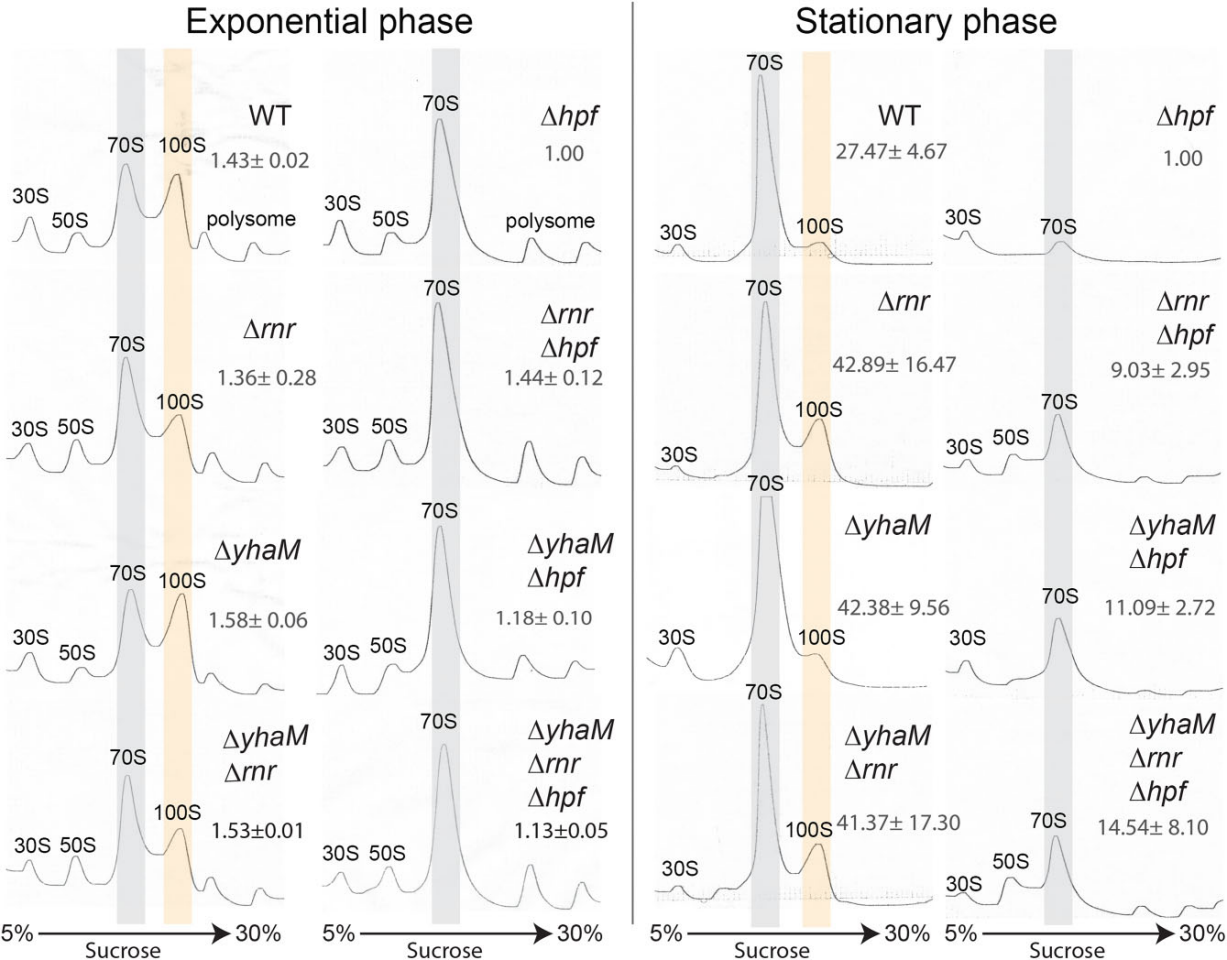
Deletion of *yhaM* and *rnr* reduces ribosome degradation in the *S. aureus* cells lacking Hpf. Ribosome sedimentation profiles of the WT, *Δrnr*, *ΔyhaM* and Δ*hpf* strains and their single, double, and triple mutants during the exponential growth (∼4.5 h at 37°C in TSB) and stationary phase (18 h at 37°C in TSB). During stationary phase, the recovery of ribosome pools occurs in a *Δrnr*Δ*hpf* (∼9-fold increase), *ΔyhaM*Δ*hpf* (∼11-fold increase), and *ΔyhaMΔrnr*Δ*hpf* (∼14-fold increase) strains compared to the Δ*hpf* mutant. A total of 2.5 Abs_260_ units of crude ribosomes was ultracentrifuged through a 5–30% sucrose gradient (x-axis), and ribosome profiles were monitored via the absorbance at 254 nm (y-axis). To obtain relative quantity of ribosomal particles (number ± SD) relative to the Δ*hpf* mutant (set as 1.0), the areas under the 30S, 50S, 70S and 100S peaks were calculated using ImageJ. ±SD indicates standard deviation from three independent replicates.

Bacterial ribosome degradation is often triggered by nutrient starvation (51–53), but a fraction of ribosomes is also degraded during exponential growth by an incompletely understood pathway (52–56). Using a methicillin-resistant *S. aureus* (MRSA) as a model, we previously showed that both Hpf-bound 70S and Hpf-dimerized 100S complexes are resistant to the 3′-5′ exoribonuclease RNase R degradation. *In vivo*, most ribosomes in an *hpf* null strain are degraded during stationary phase whereas the elimination of RNase R (encoded by *rnr*), partially restores the mature ribosome population (31). In a systematic deletion analysis of all 22 annotated RNase genes in *S. aureus*, we fortuitously found that inactivation of a cryptic exoribonuclease YhaM (formerly called Cbf1 (57)) also significantly suppressed the loss of ribosome content in the Δ*hpf* mutant during stationary phase, as indicated by the accumulation of 70S ribosomes in the Δ*yhaM* single mutant and a partial rescue of the mature ribosomes in the Δ*yhaM*Δ*hpf* double mutant relative to the Δ*hpf* mutant. A combination of *rnr* and *yhaM* deletion enhanced ribosome stability of the Δ*hpf* mutant by ∼14-fold (Figure 1). The additive effects between RNase R and YhaM imply that these RNases act on separate pathways to destabilize ribosomes. The abundance of 100S ribosomes decreases in stationary phase relative to the logarithmic growth is in part due to the disassembly of 100S complexes into 70S and subunits (20,37). Ribosome sedimentation profiling showed that approximately 50% of the mature ribosomes were preserved in a triple Δ*rnr*Δ*yhaM*Δ*hpf* knockout relative to the WT (Figure 1), implying the involvement of yet another unknown RNase or protease that facilitates ribosome degradation in the absence of Hpf.

### YhaM is widespread in gram-positive bacteria but is only present in some gram-negative bacteria and archaea

**Figure 2.**
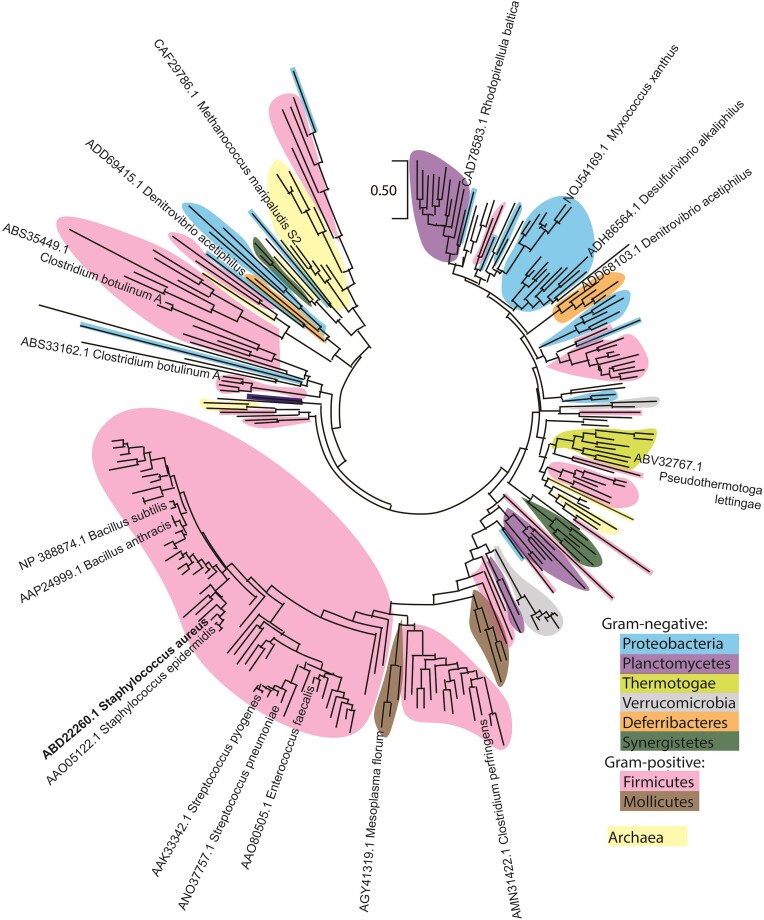
Phylogenetic tree of YhaM homologs. The tree is based on 258 homologs from 234 representative organisms obtained from database of clusters of orthologous genes (COGS). (https://www.ncbi.nlm.nih.gov/research/cog/cog/COG3481/#) (41). Representative species are labeled with their corresponding GenBank accession numbers. A full list of YhaM homologs and species are shown in Dataset 1.

YhaM homologs were initially discovered as DNA-binding proteins potentially involved in DNA replication (57,58). *In vitro*, *Bacillus subtilis* YhaM can degrade ssRNA, ssDNA and nanoRNA with high dependency on Mn(II) cations, whereas *in vivo*, YhaM can partially substitute for a loss of tRNA and rRNA processing enzymes (59–63). *Streptococcus pyogenes* YhaM degrades on average 3 nucleotides (nt) of the 3′-mRNA fragments after endoRNase cleavages (64,65). Paradoxically, *S. pneumoniae* YhaM nibbles a few nucleotides of the sRNAs and stabilizes them (66). Knowledge about the protein assembly, RNA interaction, native substrates, and physiological function of YhaM is limited. We first sought to investigate the distribution and conservation of YhaM across all domains of life. We extracted 287 YhaM homologs from 255 organisms in the Clusters of Orthologous Genes (COGs) database (41) that contains complete genomes of 1187 bacteria and 122 archaea. We built a phylogenetic tree from 258 YhaM sequences after removal of redundant sequences and repeated species. Eighty-two percent of the species contain a single YhaM, 22 of them carry two YhaM paralogs (e.g. *Clostridium botulinum* and *Bacillus haodurans*) and *C. acetobutylium* carries three copies of YhaM. YhaM homologs are commonly found in Firmicutes (∼50%) and occasionally found in Proteobacteria, Planctomycetota and archaea (Figure 2, Dataset 1). The YhaM homologs have an average length of 325 amino acids, and all possess characteristic N-terminal oligosaccharide-oligonucleotide-binding (OB) folds and the invariant His-Asp (HD) catalytic sites at their C-termini (Supplementary Figure S1). Additionally, most genomes that carry *yhaM* also carry *hpf*, implying possible coevolution of the two genes.

### *S. aureus* YhaM is not associated with the mature ribosomes

To determine if YhaM directly targets ribosomes, analogous to the RNase R (31,32). We analyzed the potential binding of YhaM to the mature ribosomes by performing sucrose density gradient fractionation followed by Western blotting. The quality of fractionation was monitored by the distribution of the 30S ribosomal protein S11. YhaM was found only in the ribosome-free low-density fractions in the WT strain. To exclude the possibility that Hpf occupancy might sterically occlude YhaM binding, the same experiments were repeated in a Δ*hpf* knockout. No YhaM was co-sedimented with ribosomes (Supplementary Figure S2). YhaM is unlikely to be a ribosome binder, but we could not exclude the possibility that YhaM may transiently interact with ribosomes and was not captured using the current approach. It is also possible that YhaM only binds to a damaged or immature ribosome with specific YhaM-ribosome stoichiometry, such as that found in RNase M5 (67).

### Inactivation of *yhaM* impairs cell growth and increases Hpf levels

**Figure 3.**
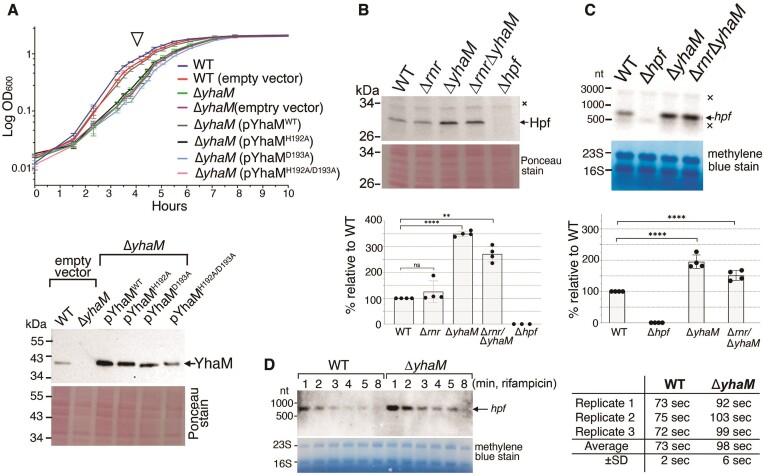
Deletion of *yhaM* causes a growth delay and the accumulation of Hpf protein and *hpf* mRNA. (**A**) An *yhaM* knockout exhibits a growth delay that can be fully complemented by a plasmid-borne *yhaM*^WT^ (denoted as pYhaM^WT^) but not its catalytically inactive mutants. Cells were grown in TSB at 37°C in three independent biological replicates. Error bars indicate ± SD. The expression levels of the endogenous YhaM and the plasmid-borne *yhaM* in the exponential phase (open arrow in the top panel) were determined by immunoblotting (lower panel). (**B**, **C**) Deletion of *yhaM* increases steady-state Hpf and *hpf* transcripts by at least 3-fold and 2-fold, respectively. The *rnr* mutant does not affect *hpf* expression. TSB cultures were harvested at OD_600_= 0.8. Nonspecific bands were marked by a crossmark. The intensity of western blot signals (Panel B) and northern blot signals (Panel C) were quantitated by ImageJ followed by paired *t* test analysis in GraphPad Prism software. ns, not significant; **P < 0.01; ****P < 0.0001. (**D**) Measurement of *hpf* mRNA half-life by rifampicin chase (400 μg/ml) followed by northern blot. Representative northern blot from three biological replicates showing slower degradation of *hpf* mRNA in the Δ*yhaM* mutant (*t*_1/2_= 98 ± 6 s) than the WT ((*t*_*1*/2_= 73 ± 2 s).

A *yhaM* mutant of *S. pyogenes* is cold-sensitive (64). While the *S. aureus* Δ*yhaM* mutant did not exhibit cold sensitivity, we observed a growth delay of the Δ*yhaM* mutant under standard laboratory conditions. The growth delay could be fully rescued by complementation of the WT *yhaM* from a plasmid but not by its catalytically compromised H192A, D193A, and H192A/D193A mutant variants (Figure 3A), suggesting that YhaM plays an important role in general RNA metabolism. A slower growth rate in the *ΔyhaM* mutant is likely due to the perturbation of RNA homeostasis beyond influencing the *hpf* transcript.

*S. aureus yhaM* is expressed during both the exponential and stationary phases (Supplementary Figure S3A). The fact that YhaM is not cofractionated with ribosomes prompted us to speculate that YhaM may modulate the levels of Hpf, thereby affecting the abundance of RNase R-resistant hibernating ribosomes. We found that strains with the Δ*yhaM* backgrounds indeed accumulated Hpf protein by at least 3-fold compared to the WT and a *rnr* mutant (Figure 3B). The increase in steady-state Hpf levels was corroborated by a ≥2-fold increase in the steady-state *hpf* transcripts (Figure 3C). The mRNA half-life of *hpf* in the WT strain was 73 ± 2 s as measured by the rifampicin chase experiments, similar to that of *S. aureus* N315 (63.7–70 s) grown in the MHI media (68). The *hpf* half-life in the Δ*yhaM* mutant was prolonged to 98 ± 6 s (Figure 3D). These results indicate that YhaM destabilizes *hpf* mRNA *in vivo*.

### Biochemical characterization of *S. aureus* YhaM

**Figure 4.**
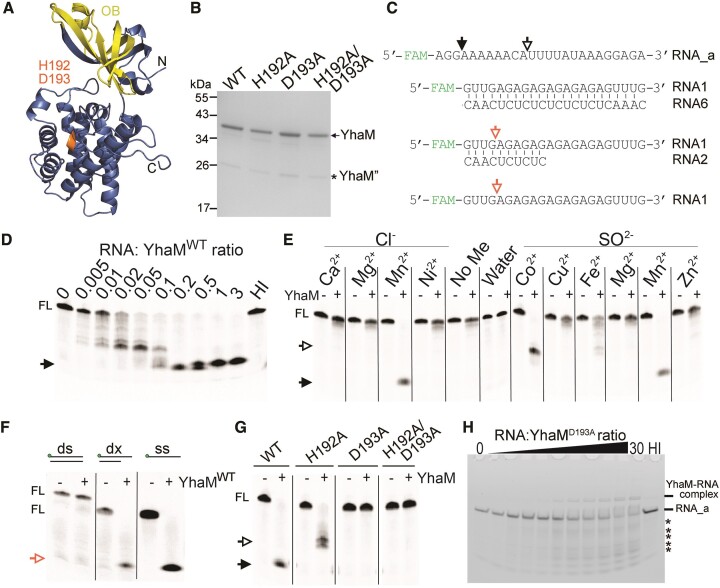
Biochemical properties of *S. aureus* YhaM. (**A**) Structural prediction of YhaM (GenBank ABD22260.1) by AlphaFold (91). The H192-D193 residues and OB-fold are colored in orange and yellow, respectively. A hexahistidine affinity tag was introduced at the N-terminus. (**B**) Analysis of purified YhaM recombinant proteins on a 4–20% SDS-PAGE and stained with GelCode™. An asterisk marks the truncated YhaM’ that has no RNase activity (60). (**C**) Sequences of 5′-fluorescently labeled RNA substrates used for the *in vitro* degradation assays. An arrow indicates the cleavage site. FL, full-length substrate. (**D**) Denaturing PAGE gel (15% TBE/urea) showing the optimal RNA-protein molar ratio (5:1) for the *in vitro* degradation of RNA_a. HI, heat-inactivated YhaM^WT^ at 95°C for 10 min. A solid arrow marks the final degraded product. (**E**) Metal selectivity of YhaM^WT^ activation. YhaM efficiently cleaves RNA_a (RNA:YhaM = 5:1) in the presence of 1 mM Mn(II) and Co(II) and to a much lesser extent in the presence of Fe(II). No difference was observed between chloride and sulfate salts. Closed and open arrows indicate full and partial cleavage intermediates, respectively. No_Me, no metal control. (**F**) YhaM^WT^ degrades ssRNA (ss, RNA_a) and RNA duplexes with 3′-overhangs (dx) but fails to degrade dsRNA (ds). A red arrow marks the cleaved product. (**G**) YhaM^D193A^ and YhaM^H192A/D193A^ proteins are catalytically inactive, whereas YhaM^H192A^ has some residual activity in digesting RNA_a. (**H**) Electrophoretic mobility shift assay (EMSA) showing that YhaM^D193A^ is RNA-binding proficient but at high concentrations can partially degrade RNA_a. HI, heat-inactivated YhaM^WT^. *****, degraded products. Samples were analyzed on a 20% TBE native gel. Fluorescence signals were visualized on an iBright™ FL1500 imager.

To assess the substrate selectivity and catalysis of YhaM on *hpf* mRNA and possibly the ribosomes, we first sought to characterize the biochemical properties of YhaM *in vitro*. The universally conserved HD domain in the YhaM homologs is a hallmark of metal-dependent phosphohydrolases (Figures 4A, Supplementary S1). We purified the recombinant WT YhaM protein (YhaM^WT^) and its HD domain mutants (YhaM^H192A^, YhaM^D193A^ and YhaM^H192A/D193A^) from the *E. coli* host using the N-terminal His_6_-tagging strategy and size-exclusion chromatography. All four proteins migrated at an estimated size of ∼36 kDa but all were copurified with an additional band of ∼26 kDa (Figure 4B). We confirmed by LC-MS/MS that this 26 kDa fragment corresponded to a truncated YhaM (Dataset 2). Importantly, the identical *S. aureus* YhaM intermediate was previously reported as a product of alternative translational initiation and was biochemically tested to be devoid of RNase activity (57,60).

To establish the *in vitro* RNA degradation assays, we used synthetic fluorescently labeled RNA_a as a substrate (Figure 4C, Supplementary Table S3), which was based on a native substrate of *S. pyogenes* YhaM (64). Titration experiments showed that the optimal reaction ratio of RNA: YhaM was 5:1 in the presence of manganese, which corresponded to a final concentration of 200 nM YhaM (Figure 4D). We found that YhaM activity has a strong dependency on specific divalent cations, with maximal stimulation by Mn(II) followed by Co(II), and to a much lesser extent by Fe(II). Unlike other RNases that rely on Mg(II) cofactors, Mg(II) did not promote YhaM activity (Figure 4E). The 3-mer and 10-mer cleavage products that we observed could be a result of YhaM endoribonuclease activity; for instance, dual exo- and endo-activity of RNase J1/J2 and RNase Z has been well documented (69,70). To eliminate this possibility, we installed five consecutive phosphorothioate (Pt) linkages at the 3′-end of RNA_a to impede 3′-5′ exonuclease digestion. Introducing Pt bonds successfully blocked digestion by YhaM under standard conditions, but the blockage was bypassed when YhaM was provided in 30-fold molar excess (Supplementary Figure S4). These results confirm that YhaM primarily functions as a 3′-5′ exoRNase and not an endoRNase.

Next, we compared the substrate specificity of YhaM using ssRNA, dsRNA, and RNA duplexes with 3′-overhangs. The RNA duplex is extremely susceptible to RNase R (31). We found that YhaM was ineffective in digesting dsRNA but could cleave the ssRNA and RNA duplex to 3–4 oligomers, suggesting that YhaM preferentially cleaves RNA substrates with a single-stranded docking site (Figure 4F). As expected, the HD domain mutants were impaired in degrading ssRNA, although the YhaM^H192A^ variant retained some activity (Figure 4G). We tested whether the inability of the YhaM^D193A^ variant to digest ssRNA was due to reduced RNA binding. Electrophoretic mobility shift assay (EMSA) showed that YhaM^D193A^ was capable of RNA binding, but at high concentrations (>10-fold excess), it also degraded the substrate (Figure 4H). Taken together, these *in vitro* experiments confirmed that the HD domain and Mn(II) are indispensable for YhaM activity and that YhaM preferentially cleaves ssRNA with 3′-5′ polarity without an apparent sequence preference.

### *hpf* mRNA is a native substrate of YhaM

**Figure 5.**
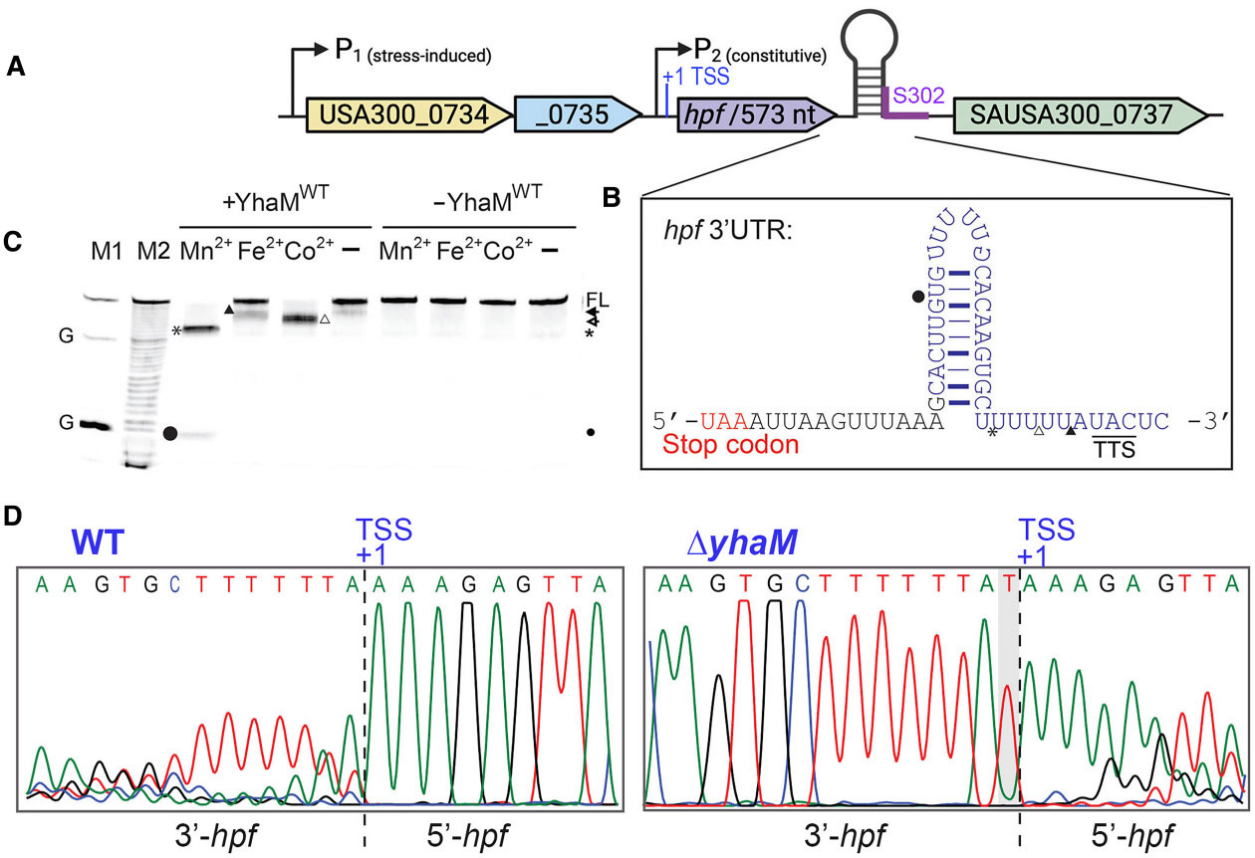
YhaM targets the 3′-*hpf* mRNA *in vitro* and *in vivo*. (**A**) Organization of *hpf* relative to the flanking genes. A hairpin marks the rho-independent transcriptional terminator (intrinsic terminator). The transcription of *hpf* is primarily driven by the P_2_ promoter. An 89-nt sRNA S302 encompassing the stem region of the hairpin terminator is marked by a purple line. TSS, transcription start site. (**B**) Zoomed-in illustration of the secondary structure and sequence of the *hpf* intrinsic terminator. Predicted transcription termination site (TTS) is underlined according to the average reported 8–10 nt extension downstream of the G-C hairpin close to the U-tract (92,93). YhaM cleavage sites are indicated on 5′-FAM RNA_c. (**C**) *In vitro* degradation of 3′-*hpf* region. Purified YhaM^WT^ cleaves RNA_c within the U-rich region (asterisk) and the hairpin loop (solid circle) in the presence of Mn(II). Varying degrees of YhaM^WT^-mediated degradation were observed in the presence of Fe(II) (solid triangle) and Co(II) (open triangle). FL, full-length RNA_c. M1, RNase T1 digested marker; M2, marker generated by alkaline hydrolysis. FL, full-length. (**D**) Sequencing chromatograms of *hpf* 5′- and 3′ regions showing the *in vivo* YhaM cleavage of *hpf* mRNA. Circular RACE mapping of the 5′- and 3′-ends revealed a 1-nt extension ‘U’ of the 3′-*hpf* in the Δ*yhaM* mutant. +1 indicates the transcription start site (TSS).

*hpf* is the last gene encoded in a 3-gene operon that carries a stress-induced SigB-dependent promoter, a constitutive CodY-dependent promoter, and a classical rho-independent terminator in its unusually long 3′-UTR (Figure 5A, B). The expression of *hpf* is primarily driven by the CodY-dependent P_2_ promoter with the transcription start site (TSS) previously mapped to an ‘A’ that lies 38-nt upstream from the start codon (36). Many targets of *S. pyogenes* YhaM possess an intrinsic terminator, with the YhaM cleavage sites mapped a few nucleotides downstream of the secondary RNA structure and U-rich tract (64). Having established the optimal *in vitro* YhaM digestion conditions, we asked whether the *hpf* intrinsic terminator hairpin could serve as a roadblock to impede YhaM processivity. A 5′-fluorescently labeled *hpf* mRNA carrying the hairpin structure was programmed with YhaM^WT^ and various divalent cations. Two intermediates corresponding to a cleavage within the U-rich tract and a cleavage within the hairpin loop were detected in the presence of Mn(II), suggesting that a secondary structure could partially block YhaM processivity *in vitro* (Figure 5C), whereas unstructured ssRNA is efficiently degraded (Figures 4D–G).

To identify the YhaM cleavage site on the *hpf* mRNA *in vivo*, we isolated RNA from both the WT and Δ*yhaM* mutant grown during logarithmic and stationary phases and performed circular RACE to map the 5′ and 3′ ends of *hpf* mRNA (49). We were able to precisely locate the + 1 transcription start site of *hpf* (36), confirming the 5′ intactness of the *hpf* transcript. Surprisingly, we consistently observed one nucleotide extension in the *hpf* 3′ extremity in the Δ*yhaM* mutant compared to the WT (Figure 5D). A 2-fold increase in cellular YhaM by expressing *yhaM* from a plasmid did not improve the 3′-end degradation (Supplementary Figure S3B). These results suggest that YhaM is an extremely low-processive enzyme *in vivo* and only removes 1 nt following the intrinsic terminator (see ‘Discussion’), whereas its processivity is much higher *in vitro* with the purified components.

### YhaM alone cannot degrade the mature ribosome *in vitro*

**Figure 6.**
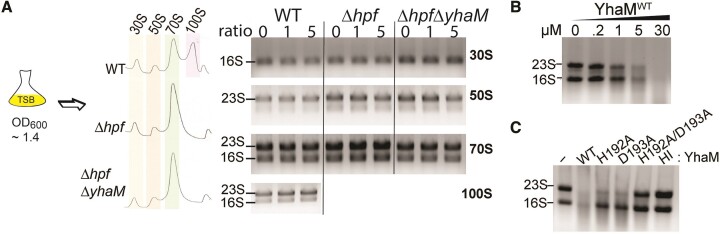
YhaM degrades rRNA at high protein concentrations but is unable to degrade mature ribosome complexes. (**A**) Cell-free ribosome degradation assays confirm that YhaM^WT^ is inactive in degrading mature 30S, 50S, 70S and 100S complexes isolated from various genetic backgrounds. Reactions were performed at a 1:1 and 1:5 ribosome-to-protein molar ratios. rRNAs were extracted from the reactions and analyzed on a 0.8% TAE agarose gel and stained with ethidium bromide. The experimental workflow is shown on the left. (**B**) At high concentrations, YhaM^WT^ degrades free 16S and 23S rRNA *in vitro*. (**C**) YhaM^WT^ degrades 16S and 23S rRNA at a concentration slightly higher than the estimated physiological concentrations (Supplementary Figure S3B, C). However, at the same concentrations, YhaM^H192A^ and YhaM^D193A^ only partially degrade the rRNAs, whereas the YhaM^H192A/D193A^ double mutant and a heat-inactivated (HI) YhaM^WT^ have no effect on rRNA degradation.

Since we could not completely rule out the possibility that YhaM may transiently bind and degrade mature ribosomes (Supplementary Figure S2), we incubated individual ribosomal subunits and complexes with 5–10-fold higher concentrations of YhaM^WT^ than the standard degradation assays. Mature ribosomes were prepared from exponentially grown WT, Δ*hpf* and Δ*hpf*Δ*yhaM* and purified by sucrose gradient density fractionation. Cell-free ribosome degradation assays were performed as described (31). YhaM^WT^ was unable to degrade any fully assembled ribosomal complexes, even in the Δ*hpf* strains without ribosome protection by Hpf (Figure 6A), confirming that mature ribosomes are not the targets of YhaM.

We next investigated whether YhaM could degrade free rRNAs using the same *in vitro* degradation assays. Total RNA was extracted from WT *S. aureus* and exposed to an excess amount of YhaM proteins. At a concentration (6 μM) slightly higher than the estimated physiological concentrations of 3.4–4.7 μM in *S. aureus* (Supplementary Figure S3B) (71), both 23S and 16S rRNA could be degraded by YhaM^WT^, with greater degradation found in the 23S rRNA (Figures 6B, C). The single HD domain mutants only partially digested 23S rRNA and the double HD mutant was completely inactive. The control experiments using the YhaM^WT^ reactions without Mn(II) (Figure 4) and the catalytically inactive YhaM^H192A/D193A^ indicate that YhaM preparation were not contaminated with RNase when provided at higher concentrations (Figure 6C). The fact that YhaM can degrade 23S rRNA is not surprising because it has been reported that *B. subtilis* YhaM has a minor role in the 3′-end maturation of 23S rRNA (62). Our findings indicate that in addition to degrading *hpf* mRNA, high cytoplasmic YhaM may spuriously degrade rRNAs.

We previously showed that in the absence of Hpf, helices h37, 41 and h44 of the *S. aureus* 16S rRNA are sensitive to ribonuclease decay, and a h37 cleaved intermediate accumulates in a *rnr* mutant (31). We used the same primer extension mapping approach to compare 16S rRNA cleavage in the presence and absence of individual YhaM and RNase R or both. Total rRNA was extracted from the mature 70S ribosome fraction after sucrose density gradient ultracentrifugation and subjected to reverse transcription. A truncated cDNA is usually indicative of a decay intermediate. Using 9 oligos to cover > 90% of the 1555-nt-long 16S rRNA, we found that the RNase-sensitive sites were almost identical to those found in our previous study, except for h3 and h32. The truncated h3 and h32 intermediates significantly accumulated in the *yhaM* and *rnr* deletion backgrounds (Supplementary Figures S6 and S7, Supplementary Table S1), suggesting both a direct and an indirect nucleolytic action of YhaM and RNase R within these regions.

### YhaM forms an active hexamer, and hexameric assembly is stimulated by Mn(II)

**Figure 7.**
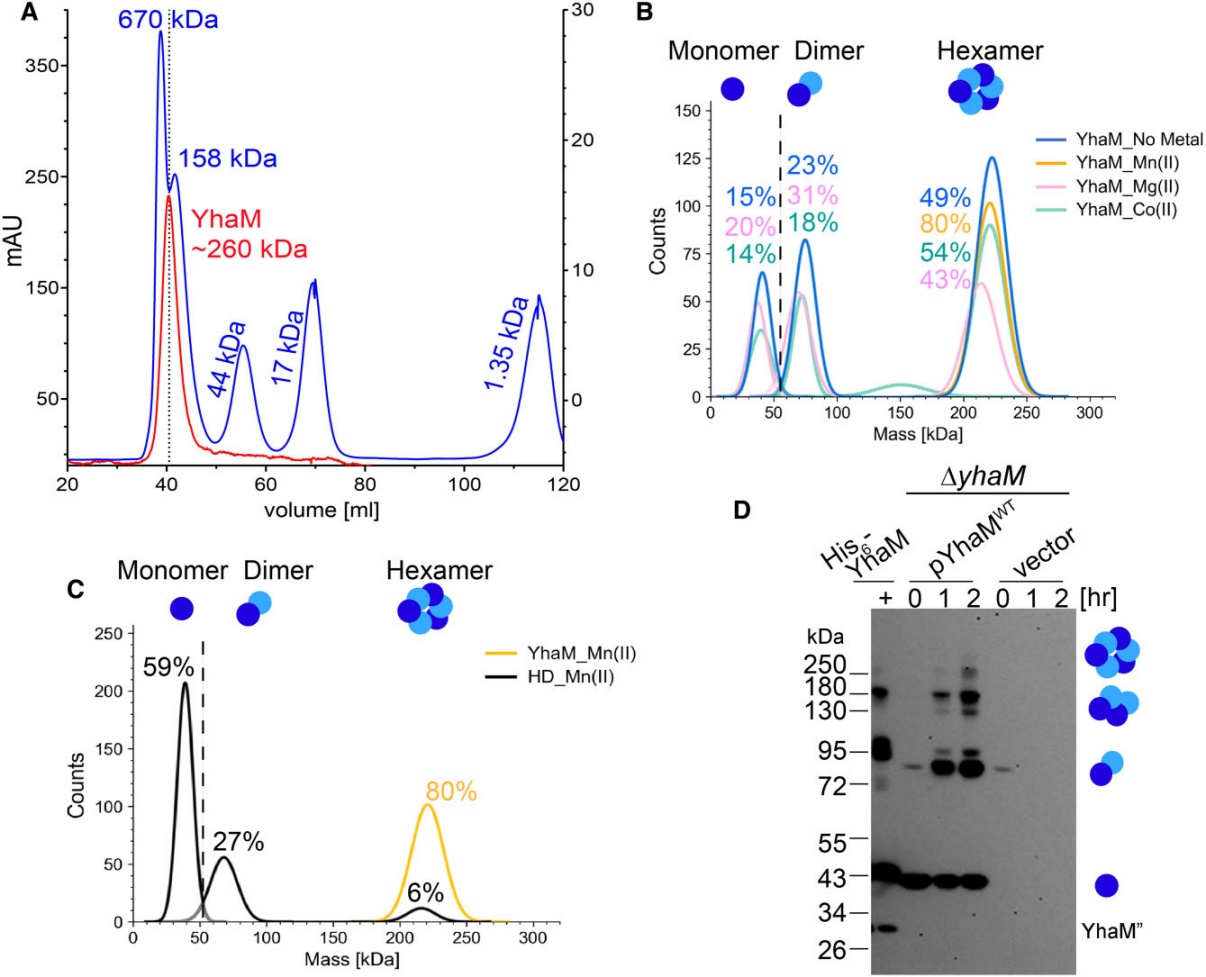
YhaM forms higher-order oligomers. (**A**) Gel filtration profile of affinity purified YhaM^WT^ on a HiPrep^TM^ 16/60 Sephacryl S-100 HR column (red trace) showing a potential hexameric assembly of YhaM^WT^. The gel filtration standard is shown in blue. (**B**) Molecular mass distribution (x-axis) of YhaM^WT^ oligomers as a function of the number of single molecules (y-axis). YhaM^WT^ exists in monomers, dimers, and hexamers. Mn(II) has the greatest (∼80%, in yellow) stimulation of promoting the formation of hexamer, followed by Co(II) (in cyan). In contrast, Mg(II) (pink) inhibits hexamer formation compared to the no metal control (blue). (**C**) The YhaM^H192A/D193A^ mutant (denoted as HD, in black) is severely impaired in oligomerization relative to YhaM^WT^ (in yellow) in the presence of Mn(II). (**D**) *In vivo* chemical crosslinking validates the dimeric and hexameric structures of YhaM^WT^. A tetramer was also observed. The *S. aureus* Δ*yhaM* mutant cells (OD_600_= 0.8) carrying an empty vector or pYhaM^WT^ were subjected to 1 mM glutaraldehyde crosslinking for 1–2 h, followed by Western blot detection of YhaM complexes on a 12% Bis–Tris NuPAGE minigel using anti-YhaM (1/2000 dilutions). Each lane corresponds to 75 μg of total protein. Purified His_6_-YhaM^WT^ served as a control and was treated with 0.5 mM glutaraldehyde (marked by ‘+’) for 5 min. YhaM’ represents the truncated YhaM shown in Figure 4B.

During protein purification, we observed a single peak on the size-exclusion chromatography that corresponds to a hexameric form of YhaM^WT^ (Figure 7A). To eliminate the possibility of protein aggregation, we collected the ∼260 kDa fraction and confirmed that the YhaM hexamer was as active as the affinity purified YhaM on the synthetic RNA_a (Supplementary Figure S5A). We also performed mass photometry on label-free YhaM variants to determine the oligomeric states (indicated by molecular mass distributions) of single molecules in solution (72). Monomers, dimers, and hexamers of YhaM^WT^ were detected by mass photometry, implying that a trimer of dimers could be the building block of the hexameric assembly. In agreement with the metal dependency of nucleolytic activity (Figure 4E), hexamers were prevalent in the presence of Mn(II) and Co(II), but the formation was inhibited by Mg(II). As many as 80% of the particles were in a hexameric state in solution with Mn(II) (Figure 7B). Mutations in the HD domain strongly reduced the multimeric assembly even in the presence of Mn(II) (Figures 7C, S5B, C), suggesting that the HD domain also plays a role in oligomerization.

To test whether YhaM can exist as a hexamer in cells, we expressed YhaM^WT^ in the Δ*yhaM* mutant on a plasmid under the control of its native promoter. The expression levels of plasmid-borne YhaM were 2-fold greater than those of endogenous YhaM (Supplementary Figure S3B). Exponentially grown *S. aureus* cells were subjected to chemical crosslinking with glutaraldehyde, a nonzero length crosslinker. Immunoblotting showed the presence of monomers, dimers, tetramers, and hexamers in cells, similar to that found in the recombinant His_6_-YhaM control (Figure 7D). Notably, the tetramers were not detected by mass photometry, presumably because glutaraldehyde was able to capture the transient formation of a tetramer. The relative quantity of each multimer could not be accurately determined due to the varying transfer efficiencies of the complexes during immunoblot transfer. In summary, the ability to detect the YhaM hexamer in cells confirmed that the oligomers observed by size-exclusion chromatography and mass photometry are not artifacts and that YhaM predominantly exists as a dimer and a hexamer in live cells. Additionally, the low *in vivo* processivity of YhaM (Figure 5D) may be partially influenced by the cellular concentrations and/or multimeric state of YhaM (see ‘Discussion’).

## Discussion

**Figure 8.**
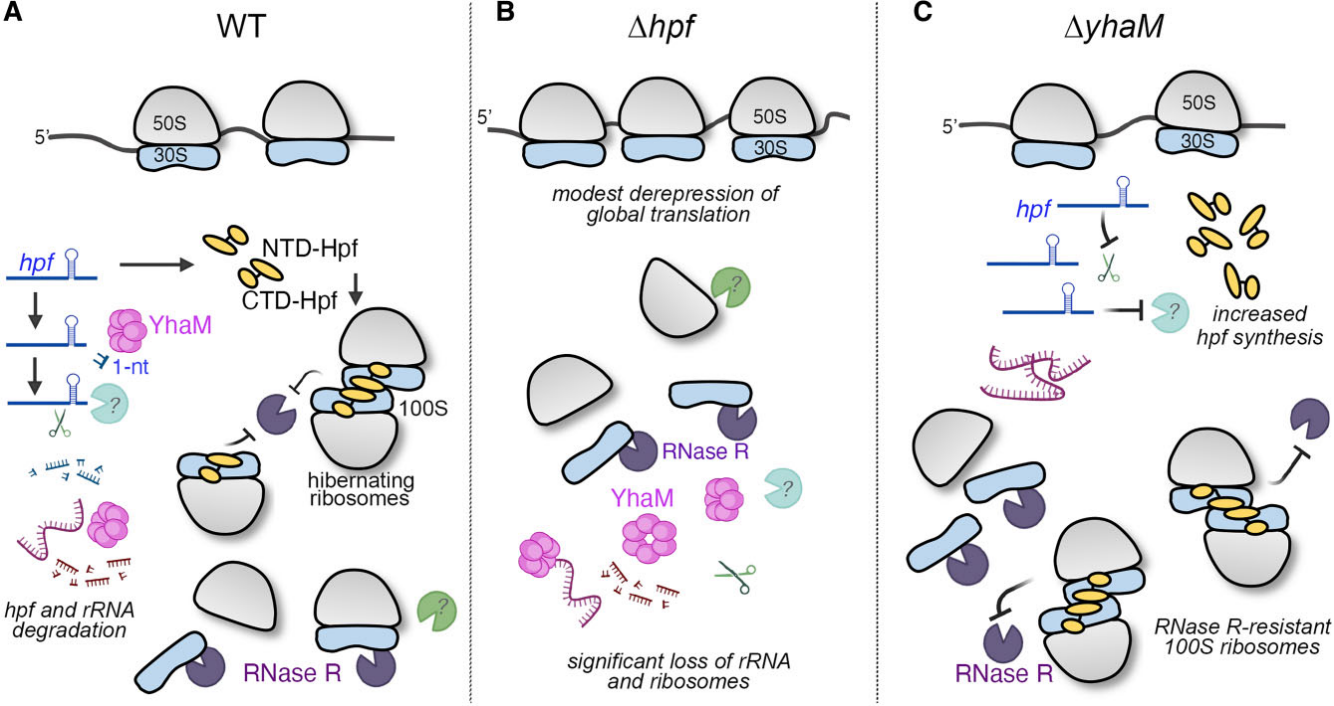
A model for ribosome degradation in *S. aureus*. (**A**) In the WT cells, Hpf binding to 70S and 100S ribosomes occludes the nucleolytic action of RNase R, which targets h37, h41 and h45 of the 16S rRNA (31). The Hpf C-terminal domain (CTD) promotes self-dimerization, and the N-terminal domain (NTD) sterically prevents tRNA and mRNA binding to the ribosomes (14). The hexameric YhaM trims the 3′-*hpf* mRNA by 1-nt downstream of an intrinsic terminator and the U-rich sequence, followed by the degradation by another unidentified ribonuclease (denoted by a question mark). High cellular concentrations of YhaM also promote rRNA degradation. (**B**) In the Δ*hpf* mutant, global translation is modestly increased in the absence of 100S complexes (21), a fraction of ribosomes is exposed to RNase R (31) and other unknown proteases/RNases, leading to a significant loss of functional ribosomes. (**C**) In the Δ*yhaM* mutant, RNase R-mediated loss of ribosomes is compensated by an increase in Hpf biosynthesis, resulting in more Hpf-bound, RNase R-resistant ribosomes.

RNases are usually the direct contributors of bacterial ribosome degradation (52,54,70,73–76). For instance, *E. coli* RNase I serves as the initiator endoribonuclease, and Gram-positive and Gram-negative bacterial RNase R and YbeY function to remove faulty and premature ribosomes by digesting 16S rRNA. Here, we report a new and indirect pathway by which exoRNase YhaM in *S. aureus* destabilizes ribosomes by degrading the *hpf* mRNA, which encodes a ribosome protector. An unidentified RNase is likely involved in *hpf* decay following the 1-nt trimming by YhaM (Figure 8). Hpf-bound 70S and 100S ribosomes are resistant to RNase R cleavage (31). Hpf dimerizes the two 70S monomers, and the occupancy of Hpf and/or the ‘side-by-side’ dimeric architecture of the 70S dimer (100S complex) presumably sterically blocks the binding of RNase R and other unknown RNase/protease (14,31,75). Inactivation of *yhaM* thus suppresses ribosome degradation by increasing the abundance of Hpf. YhaM can degrade free rRNA at high concentrations (Figure 6), and it is possible that eliminating *yhaM* also partially increases rRNA availability to produce more mature ribosomes. The RNase or protease responsible for the degradation of a significant fraction of ribosomes in the Δ*yhaM*Δ*rnr*Δ*hpf* strain remains to be identified (Figure 1).

*S. aureus* YhaM exhibits high processivity *in vitro* but only trims the *hpf* mRNA by 1 nt *in vivo*. Short nucleotide (3–6 nt) trimming by the 3′-5′ exoRNases has been reported in other YhaM homologs (64–66,77), e.g. RNase T (74), RNase II (78), and RNase PH (61). It is unclear why YhaM stalls after nibbling 1 nt following the intrinsic terminator. The purpose of this seemingly inefficient processing requires further investigation. It is possible that *in vivo* mRNA degradation by YhaM is blocked by an RNA-binding factor or by a YhaM interactional partner. YhaM may function as a distributive enzyme in which the protein is released from its RNA substrate when each nucleotide is hydrolyzed. Intracellular Mn(II) levels can potentially modulate YhaM assembly and catalysis. Intracellular concentrations of Mn(II) in *E. coli* are in the low micromolar range (∼1.7 μM) (79). However, we found that supplementing cell cultures with 1–5 mM Mn(II) does not alter the 3′-end identity. Alternatively, the oligomerization states of YhaM may determine the fate of RNA degradation with high concentrations driving the formation of the most active hexamer (or higher RNA binding affinity) and an intermediate concentration promoting the less active dimer. While the YhaM protein can be detected from logarithmic growth to stationary phase, how the expression of *yhaM* is upregulated is unknown.

Through an unexplained mechanism, this idiosyncratic 1-nt trimming of YhaM was also observed in a global Rend-seq analysis of *B. subtilis*. Among the 339 YhaM targets, 80% of the 74 intrinsic terminator-containing transcripts have a 1-nt extension in the Δ*yhaM* mutant than that in the WT, whereas the remaining substrates had a 2–3 nt extension (78), supporting the trimming activity of YhaM. The overrepresentation of intrinsic terminators in the YhaM targetome (64,66,78) implies that the intrinsic terminator structure may serve as an important RNA recognition element or a blockage to prevent over-digestion. Curiously, a small non-coding S302 RNA of 89 nt encompassing the *hpf* intrinsic terminator has been reported in *S. aureus* (Figure 5A) (80,81). The *E. coli* sRNA chaperone Hfq adopts a homo-hexameric ring to promote target base-pairing and recruitment of RNase E to degrade sRNA-RNA hybrids (82). It is tempting to speculate that the hexameric YhaM may play a dual role as a Hfq-like chaperone and RNase to regulate sRNA-dependent decay.

No atomic structure of any apo-YhaM homologs or RNA-YhaM complex is currently available. The observation of dimeric and hexameric YhaM is surprising, as higher-order oligomers are uncommon and have been reported in only a handful of bacterial RNases, e.g. trimeric PNPase, tetrameric RNase E and RNase J, hexameric RNase PH and YloC (74,83–89). The oligomeric state of YhaM during RNA and metal binding and the sequence of order for YhaM assembly will need to be clarified at the structural and biophysical levels.

The null mutant of *S. aureus yhaM* impairs cell growth even under nutrient-sufficient laboratory conditions (Figure 3A). The growth delay can be due in part to inadequate processing of other critical RNAs (apart from *hpf* mRNA) and a loss of YhaM from the divisome (58). A Tn insertion mutant of *S. pneumoniae yhaM* is severely reduced in murine nasopharynx infections and compromised in lung colonization (90). It is likely that *S. aureus* YhaM plays a critical role in the maturation and quality control of RNAs that are involved in host interactions.

Limitations of the study. The genome-wide targetome of YhaM in *S. aureus* remains to be investigated. *S. pneumoniae* YhaM acts as a stabilizer of sRNAs involved in natural competence (66). More work is needed to discern a possible dual role of YhaM as both a stabilizer and a degrader. The precise physiological significance of 1–3 nt mRNA trimming awaits in-depth analyses. The discriminators of RNA-binding and metal selectivity, as well as the atomic structures of apo-YhaM and the YhaM-RNA complex, will offer a broader mechanistic understanding of this peculiar exoRNase.

## Supplementary Material

gkae596_Supplemental_Files

## Data Availability

All relevant data are within the manuscript and its supplementary data files.
