# Supplementary material for: Staphylococcal exoribonuclease YhaM destabilizes ribosomes by targeting the mRNA of a hibernation factor
Source: Nucleic Acids Res. 2024 Jul 9;52(15):8998–9013. doi: 10.1093/nar/gkae596 (PMC11347170; doi:10.1093/nar/gkae596)
Supplement: gkae596_Supplemental_Files [file gkae596_supplemental_files.zip › Liponska_Supplemental_revision.pdf]

SUPPLEMENTARY DATA

Staphylococcal exoribonuclease YhaM destabilizes ribosomes by targeting the mRNA of a hibernation factor

Anna Lipońska[1], Hyun Lee[2] and Mee-Ngan F. Yap[1*]

[1] Department of Microbiology-Immunology, Northwestern University Feinberg School of Medicine, 320 E Superior St, Chicago, IL 60611.

[2] Department of Pharmaceutical Sciences, College of Pharmacy and Biophysics Core in Research Resources Center, University of Illinois at Chicago (UIC), 1100 S Ashland Ave Chicago, IL 60607.

* To whom correspondence should be addressed:

Email: frances.yap@northwestern.edu | Tel: +1 (312) 503-3793

Table S1. Comparison of ribosome hibernation factors and RNases in Gram-negative *E. coli* and Gram-positive *S. aureus*.

**Ribosome and RNases**	***E. coli***	***S. aureus***
Hpf expression	Starvation and stationary phase (1).	All growth phases (2-4).
Architecture of 70S dimers	Back-to-back (5).	Side-by-side (6-8).
Hibernation factors	100S: RMF and Hpf (9-11). Inactive 70S: YfiA (RaiA) (9,12) .	Long Hpf (3,6,7).
Protected ribosomal sites on hibernating 70S and 100S.	h24, h28, h44, h45 (13).	h3, h32, h37, h41, h44 (this study) (14).
Hpf-dependent protection of ribosomes from RNases.	RNase R and YbeY (13).	RNase R (14) and YhaM (this study).
3'-5' ExoRNases (15-17)	RNase II, RNase D, RNase T, RNase PH, NrnA, NrnB.	YhaM, NrnA.
Common [a]	PNPase, RNase R.	
Endonucleases (15-18)	RNase LS, RNase G, RNase I, RNase E.	Mini-III, YhcR, RNase Y, RNase M5, RNase HIII, Rae1, RNase J.
Common [a]	YbeY, RNase III, RNase P, RNaze Z, RNase HII, RNase HI.	

[a] shared between *E. coli* and *S. aureus*.

Table S2. Bacterial strains and recombinant DNA.

Name	Genotypes and relevant features	Source
S. aureus		
JE2	Parental strain, plasmid cured LAC USA300 strain	BEI resources (19)
RN4220	*sau1*$^-$, *hsdR*$^-$, *mec*$^-$, *rsbU*$^-$, *agr*$^-$, plasmid passage host	ATCC NR-45946
MNY133	JE2 Δ*hpf*::Km	Ref (14)
NE501	JE2 Δ*rnr*::Erm	BEI resources
MR637	JE2 Δ*yhaM*::Erm	This study
MNY175	JE2 Δ*rnr*::Erm, Δ*hpf*::Km	Ref (14)
MNY176	JE2 Δ*yhaM*::Erm, Δ*hpf*::Km	This study
MNY200	JE2 Δ*rnr*::Erm, Δ*yhaM*::Spc	This study
MNY206	JE2 Δ*rnr*::Erm, Δ*yhaM*::Spc Δ*hpf*::Km	This study
E. coli		
BL21(DE3)	F$^-$ *ompT gal dcm lon hsdS*$_B$(*r*$_B^-$*m*$_B^-$) λ(DE3 [*lacI lacUV5-T7p07 ind1 sam7 nin5*]) [*malB*$^+$]$_{K\text{-}12}$(λ^S)	Lucigen
DC10B	DH10B Δ*dcm*	Ref (20)
**Plasmids** pLI50	*E. coli*-*S. aureus* shuttle vector, promoterless Amp^R, Cm^R	Addgene (21)
pLI50::yhaM	pP$_{yhaM}$-*yhaM*, *yhaM* bearing its native promoter on pLI50, Amp^R, Cm^R	This study
pLI50::yhaM H192A	*yhaM*(H192A) bearing its native promoter on pLI50, Amp^R, Cm^R	This study
pLI50::yhaM D193D	*yhaM*(D193A) bearing its native promoter on pLI50, Amp^R, Cm^R	This study
pLI50::yhaM H192A D193D	*yhaM*(H192A, D193A) bearing its native promoter on pLI50, Amp^R, Cm^R	This study
pMCSG7	Ligation independent overexpression plasmid, cleavable His tag by TEV, Amp^R.	Ref (22)
pMCSG7::yhaM (WT)	~940 bp *yhaM* on pMCSG7, Amp^R, encoding N-terminally 6His-tagged YhaM	This study
pMCSG7::yhaM (H192A)	~940 bp *yhaM*(H192A) on pMCSG7, Amp^R, encoding N-terminally 6His-tagged YhaM(H192A)	This study
pMCSG7::yhaM (D193A)	~940 bp *yhaM*(D193A) on pMCSG7, Amp^R, encoding N-terminally 6His-tagged YhaM(D193A)	This study
pMCSG7::yhaM (H192A/D193A)	~940 bp *yhaM*(H192A/D193A) on pMCSG7, Amp^R, encoding N-terminally 6His-tagged YhaM(H192A, D193A)	This study

Table S3. Oligonucleotides

Primer	Sequence (5'-3')[a,b]	Application
**DNA**		
P1440	TACTTCCAATCCAATGCCATGAGAAATATAGAGAATCTAAATC	Ligation-independent cloning of *yhaM* into pMCSG7
P1441	TTATCCACTTCCAATGTTATTAATCGAGTGATTCAGGATTGTAGA	
P457	TAATACGACTCACTATAGGG	DNA sequencing primers on pMCSG7
P458	GCTAGTTATTGCTCAGCGG	
P1692	AGTGGTATTATTTTGGCTGATATTGGTAAAGTTAGA	Mutagenesis of *yhaM* (H192A)
P1693	TCTAACTTTACCAATATCAGCCAAAATAATACCACT	
P1694	AGTGGTATTATTTTGCATGCTATTGGTAAAGTTAGA	Mutagenesis of *yhaM* (D193A)
P1695	TCTAACTTTACCAATAGCATGCAAAATAATACCACT	
P1823	AGTGGTATTATTTTGGCTGCTATTGGTAAAGTTAGA	Mutagenesis of *yhaM* (H192A/D193A)
P1824	TCTAACTTTACCAATAGCAGCCAAAATAATACCACT	
P1684	AACCTACAGAAGCTTACTGTTATAAATGTGTCTCAATTTC	Gibson assembly cloning of pP*yhaM*-*yhaM* into pLI50
P1685	TATTTCTCATACATCCATATCTCCTTAATTAAAGG	
P1686	AGGAGATATGGATGTATGAGAAATATAGAGAATCTAAATCCC	
P1687	ATGATCTTTATAATCATCGAGTGATTCAGGATTGT	
P1688	GATTATAAAGATCATGATGGCGATTATAA	
P1689	AAGCTTCTGTAGGTTTTTAGGC	
P630	GCACATTTCCCCGAAAAGTGCCACCTGACGT	DNA sequencing primers on pMCSG7
P631	TGCCTTTATTTTGAATTTTAAGGGGCAT	
P1825	GCGAATTAATACGACTCACTATAGATACAACTGGATTAACAATTCATCGTGC	T7 promotor DNA template
P1827	GAGTATAAAAAAGCACTTGTGCAAAAACACAAG	
P1828	TTCGTTCGCTCCTTTAGCTAAAAAACTGTTTG	
P1858	GCGTATAAATCATCGTTTCGCTCTTCAG	*hpf* reverse transcription
P1856	CCTCAATATAGTTGCGAATAGCATCT	Circular RACE nested PCR
P1857	CGTAAAGACGGTAAATATGGCTTG	
P1859	GAATTTGAATAAGTTTTAACTTTAACATGCGCCACT	
P1860	TGGATTCAGAAGAAGCGGTATTACAAATGAATC	
P1943	CGGCGGTAAACGATACTTGTTCCATCAG	DIG-labeled *hpf* probe
P1996	CACCGGCATTCTCACTTCTAAGCGCTCC	DIG-labeled 23S rRNA probe
**a**: 16S (1484-1506)	(6-FAM)-ACTTCACCCCAATCATTTGT	Primer extension
**c**: 16S (1198-1215)	(6-FAM)-ATGATGATTTGACGTCAT	
**g**: 16S (1329-1349)	(6-FAM)-TCCAGCTTCATGTAGTCGAGT	
**i:**16S(148-170)	(6-FAM)-AGCTCCGGTTTCCCGAAGTTATC	

**RNA** RNA_a	(6-FAM)-AGGAAAAAACAUUUUAUAAAGGAGA	*In vitro* YhaM substrate
RNA_b	(6-FAM)-AGGAAAAAACAUUUUAUAAA*G*G*A*G*A	*In vitro* YhaM substrate to block 3'-exo.
RNA_c	(6-FAM)-CACUUGUGUUUUUGCACAAGTGCUUUUUUAUACUC	*In vitro* YhaM substrate, *hpf* terminator
RNA1	(6-FAM)-GUUGAGAGAGAGAGAGAGUUUG	*In vitro* YhaM substrate[28,41]
RNA2	CAACCUCUCU	Complementary sequences to RNA1
RNA6	CAAACUCUCUCUCUCUCUCAAC	

[a]: mutagenesis sites are underlined.
[b]: phosphorothioate (pt) bonds are marked by an asterisk.

Table S4. Chemicals, peptides, and recombinant proteins.

Reagent or Resource	Source	Identifier
Sucrose	Sigma	S0389- 500G
HEPES	Sigma	H3375-500G
20×MOPS NuPAGE	Invitrogen	NP0001
50×TAE	VWR	K915-1.6L
10×Tris-glycine-SDS	ThermoScientific	J61006.K7
5XTBE	Invitrogen	LC6675
Magnesium acetate	Sigma	M0631-500G
Fluorinert FC-70	Sigma	F9880-100ML
Potassium Chloride	Sigma	P3911-500G
DTT	MIDSCI	IB21045
PMSF	Sigma	P7626-25G
Ammonium Chloride	Sigma	21330-500G
Calcium Chloride	Sigma	C1016-500G
Magnesium chloride	Sigma	208337-1KG
Manganese Chloride	Sigma	244589-10G
Nickel Chloride	Sigma	364304-100G
Cobalt Sulfate	Sigma	C6768-250g
Copper sulfate	Sigma	209198-100G
Ferrous Sulfate	Fisher	S25325
Magnesium sulfate	sigma	M7506-500G
Manganese sulfate	Alfa Aesar	B22081
Zinc Sulfate	MP Biomedicals	191452
PBS(10×)	Fischer Scientific	BP665-1
Tween 20	Sigma	P1379-500ML
TCA	Sigma	T6399-500G
Acetone	Sigma	650501-1L
Tris-HCl (pH 7.5)<br>Tris-HCl (pH 8)	VWR	E691-500ML<br>E199-500ML
Sodium azide	Sigma	438456-5G
EDTA	Amresco	E177-500ML
Imidazole	Sigma	I2399-100G
Phenol/Chloroform (pH 6.7-6.8)	BioExpress	0883-100ML
Acidic Phenol (pH 4.5)	BioExpress	0981-400 mL
Chloroform: isoamylalcohol (24:1)	BioExpress/Amresco	X205-450mL
Isopropanol	Sigma	I9516-500ML
Ethanol	Sigma	E7023-6X500ML
Sodium acetate	Alfa Aesar	J61928
Glycogen	Invitrogen	10814-010
Erythromycin	Sigma	45674-25G-F
Chloramphenicol	Sigma	C0378-25G
Kanamycin	Sigma	K1377-5G
Ampicillin	Sigma	A9518-25G
IPTG	Sigma	I5502-5G
Sodium Hydroxide	VWR	MK469360
Acetic acid	Sigma	A6283-2.5L
Ethidium bromide solution	Sigma	E1510-10ML

Urea	Sigma	U6504-500G
30 % Acrylamide/Bis Solution, 19:1	BioRad	1610154
Ammonium persulfate	Sigma	A3678-100G
TEMED	Sigma	T9281-50ML
Ponceau S Stain	VWR	K793-500ML
GelCode Blue Safe Protein Stain	Fisher Scientific	PI-24594
Tween20	Sigma	P1379-500ML
LE Quick Dissolve Agarose	GeneMate	E-3120-500
Methylene blue	Sigma	M4159-100G
20×SSC OmniPur	Calbiochem	8340-1PACK
Nickle NTA agarose	McLAB	NiNTA-300
Glycerol	Sigma	G5516-500ML
Tricine	Sigma	T0377-1KG
Bromophenol Blue	Sigma	B0126-25G
Xylene cyanol FF	Sigma	335940-10G
91% formamide	Sigma	F9037-100ML
Sodium carbonate	Sigma	223530-500G
glutaraldehyde	Sigma	G5882
RppH	New England Biolabs	M0356S
RNase H	New England Biolabs	M0297L
Lysostaphin	AMBI	LSPN-50
Turbo DNase	Life Technologies	AM1907
T4 RNA ligase I	New England Biolabs	M0204L

Table S5. Critical commercials kits.

Reagent or Resource	Source	Identifier
HiFi PCR Premix	Takara	639298
DreamTaq Green PCR Master Mix	Thermo Scientific	K1081
Gibson Assembly Kit	New England BioLabs	E2611L
Thermo Sequenase Cycle Sequencing	Affymetrix	78500
SuperScript III Reverse Transciptase	Invitrogen	18080-044
HighYield T7 Fluorescein RNA labeling	Jena Bioscience	RNT-101-FAMX
DIG Northern Starter Kit	Roche	12039672910
Quick Start™ Bradford Protein Assay	BioRad	5000205
SuperSignal™ West Dura	Thermo Scientific	34076
Gel Filtration Standard	BioRad	151-1901
QuikHyb hybridization solution	Agilent Technologies	201220
ULTRAhyb hybridization buffer	Invitrogen	AM8670
Halt™ protease Inhibitor cocktail	Thermo Scientific	78437

Table S6. Antibodies.

Reagent or Resource	Source	Identifier
Anti-YhaM	Pacific Immunology	This Study
Anti-S11	Yap lab collection	Ref (23)
Anti-Hpf	Yap lab collection	Ref (23)
HRP-conjugated anti-IgG	Cytiva	NA9120
Alexa Fluor Plus 800 anti-Rabbit IgG	Invitrogen	A32735

Table S7. Others.

Reagent or Resource	Source	Identifier
RNeasy mini kit	Qiagen	74104
Gel/PCR DNA Fragments Extraction Kit	IBI Scientific	IB41030
High-Speed Plasmid Mini Kit	IBI Scientific	IB47102
mini nitrocellulose membrane turbo stack	Bio-Rad	1704158
Mini PVDF membrane	Bio-Rad	1704156
15% TBE-Urea Gels 1mm 12 well	Invitrogen	EC68852BOX
20% TBE-native gel	Invitrogen	EC63152BOX
4-12% Bis-Tris NuPAGE minigels	Invitrogen	NP0323BOX
4-20% TGX SDS-PAGE	Bio-Rad	4561096
Amicon Ultra MWCO-10	Millipore Sigma	UFC801024
iBright FL1500 system	ThermoScientific	
FastPrep-24 homogenizer	MP Biomedicals	
Stratalinker™	Agilent	
Nanodrop	ThermoScientific	
HiPrep™ Sephacryl S-100 HR 16/60	Cytiva	
TwoMP Mass photometer	(Refeyn Ltd, Oxford, UK).	

Experimental models
Staphylococcus aureus USA300_FPR3757

Software and algorithms
Scaffold Software
Mega 11JAlview
Adobe Illustrator
GraphPad Prism v9
ImageJ
FinchTV
Acquire MP software
DiscoverMP (Refeyn)

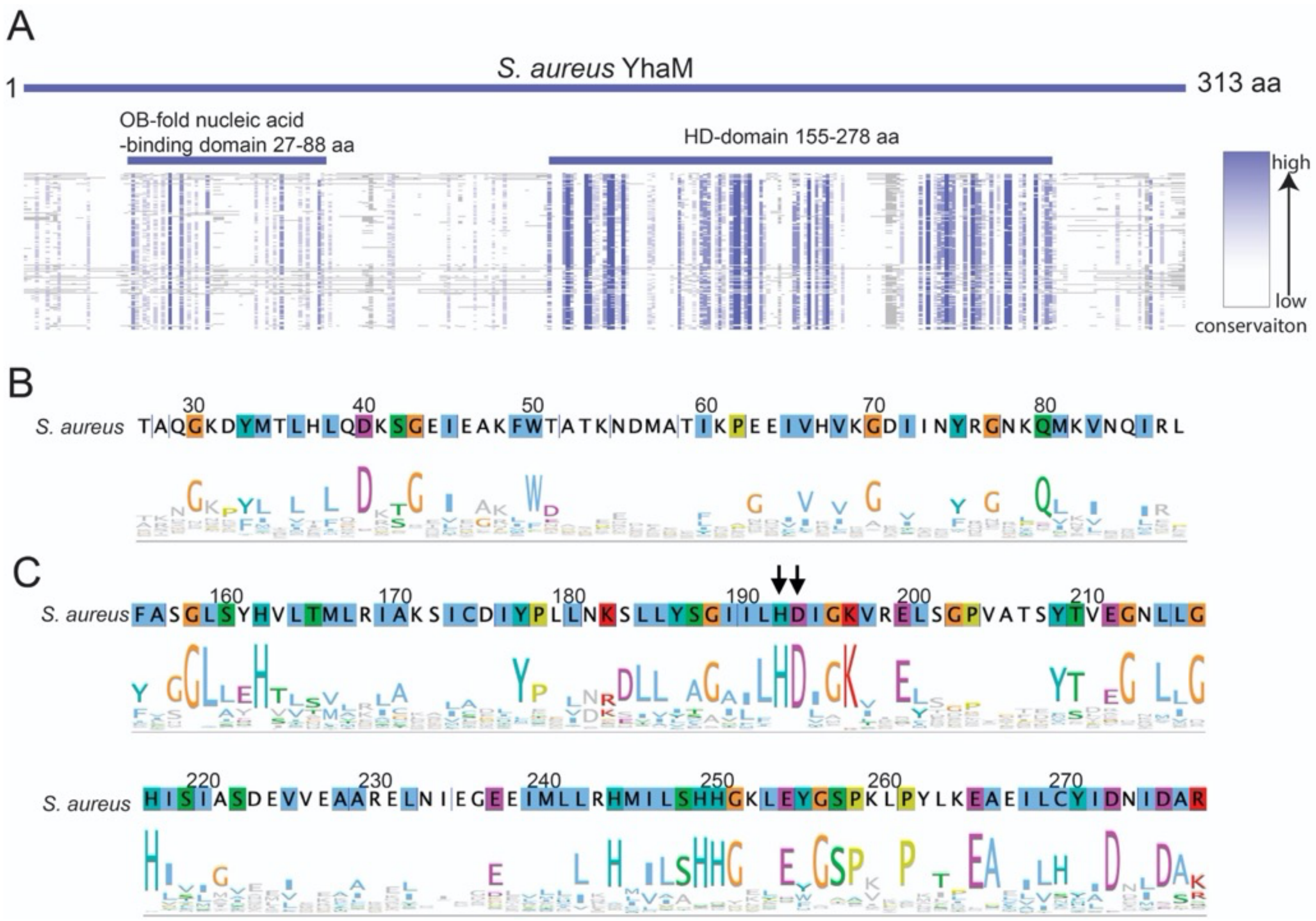

Figure S1. **Conservation of YhaM homologs. (A)** Multiple-sequence alignment of representative 258 YhaM homologs. The alignment was performed using MUSCLE algorithm. The degree of conservation is illustrated in violet color gradient. The OB-fold nucleic acid-binding domain (27-88 a.a.) and HD-domain (155-278 a.a.) of *S. aureus* YhaM are marked. **(B)** Graphical representation of sequence conservation within the OB-fold nucleic acid-binding domain. **(C)** Sequence conservation of YhaM HD-domain. His-Asp residues (solid arrows) are invariant in all YhaM homologs.

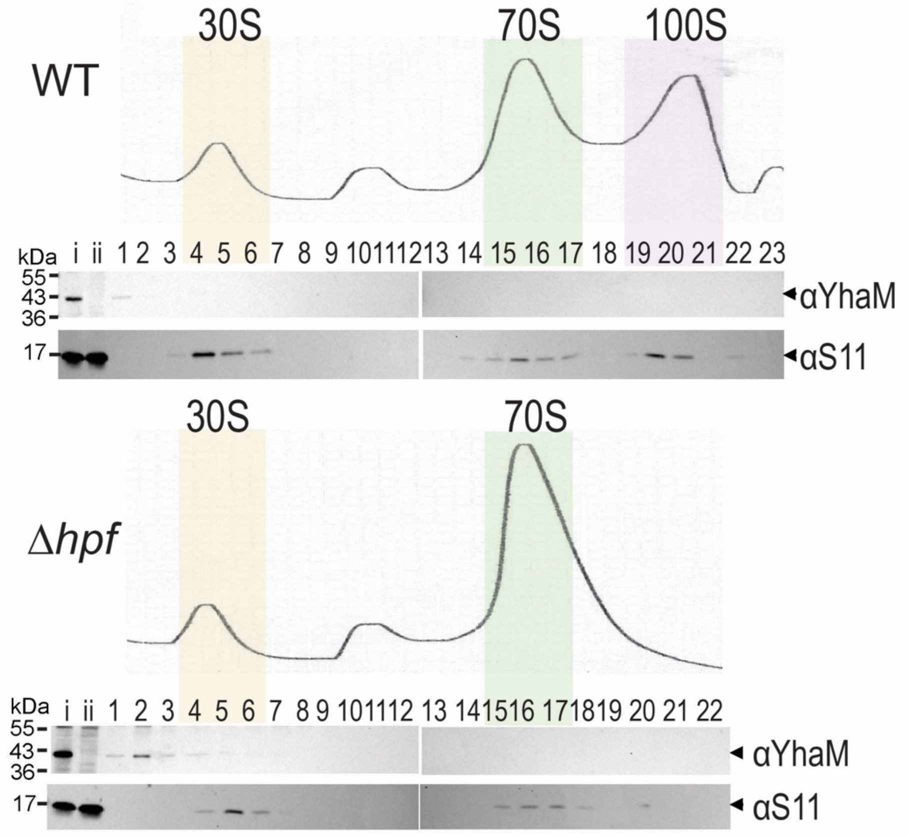

Figure S2. ***S. aureus*** **YhaM does not co-sediment with the mature ribosomes in both the WT and ∆*hpf* strains**. Crude ribosomes isolated from late exponential phase cells were ultracentrifuged through a 5-30% sucrose density gradient (x-axis) and monitored by Abs_{254} (y-axis). Each fraction was precipitated by a final concentration of 10% TCA, resolved on 4-20% SDS-PAGE and probed with anti-YhaM (1/2,000) and anti-S11 (1/4,000) antibodies. Total lysates from the WT (lane "i") and ∆*yhaM* (lane "ii") served as a control for antibody specificity. The 30S ribosomal protein S11 serves as a fractionation marker.

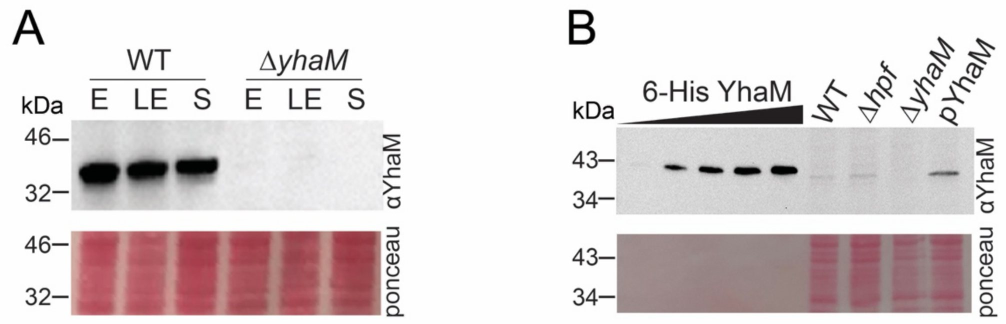

C

S. aureus	YhaM concentrations (ng/μg)[a]	*S. aureus* COL[b]	
WT	0.1249 ± 0.07 (~4.69 μM)	YhaM (ng/μg)	0.0917 (~3.44 μM)
Δ*hpf*	0.1699 ± 0.07 (~6.27 μM)	Hpf (ng/μg)	1.0104 (~62.06 μM)
Δ*yhaM*(pYhaM)	0.2306 ± 0.07 (~8.66 μM)		

[a] *This study, n=3*
[b] *previous published label free quantitative proteomics, Maass et al. (2011)*

Figure S3. Expression and abundance of YhaM in *S. aureus*. (A) YhaM is expressed during all growth phases in *S. aureus*. Western blot showing the expression of YhaM during the early exponential (E) (OD_{600}=0.8, ~2 hr growth), late exponential (LE) (4-4.5 hr growth) and stationary phase (S) (18-20 hr growth). Ponceau stain serves as a loading reference. **(B)** Estimation of cellular YhaM by Western blots. A range of 10-20 ng recombinant His_6-tagged YhaM protein was used as a standard. Seventy-five micrograms of total proteins from the WT, Δ*hpf,* Δ*yhaM* and Δ*yhaM* (pYhaM^{WT}) strains from exponential grown cells (OD_{600}=0.8) were loaded per lane. **(C)** A summary of YhaM abundance from 3 independent biological replicates in panel B. The results are comparable to the published report (24) (right column). The cellular concentrations of Hpf (22 kDa) and YhaM (36 kDa) in *S. aureus* have been previously determined using a label-free proteomics approach (24). Molarity was calculated based on published equations (25).

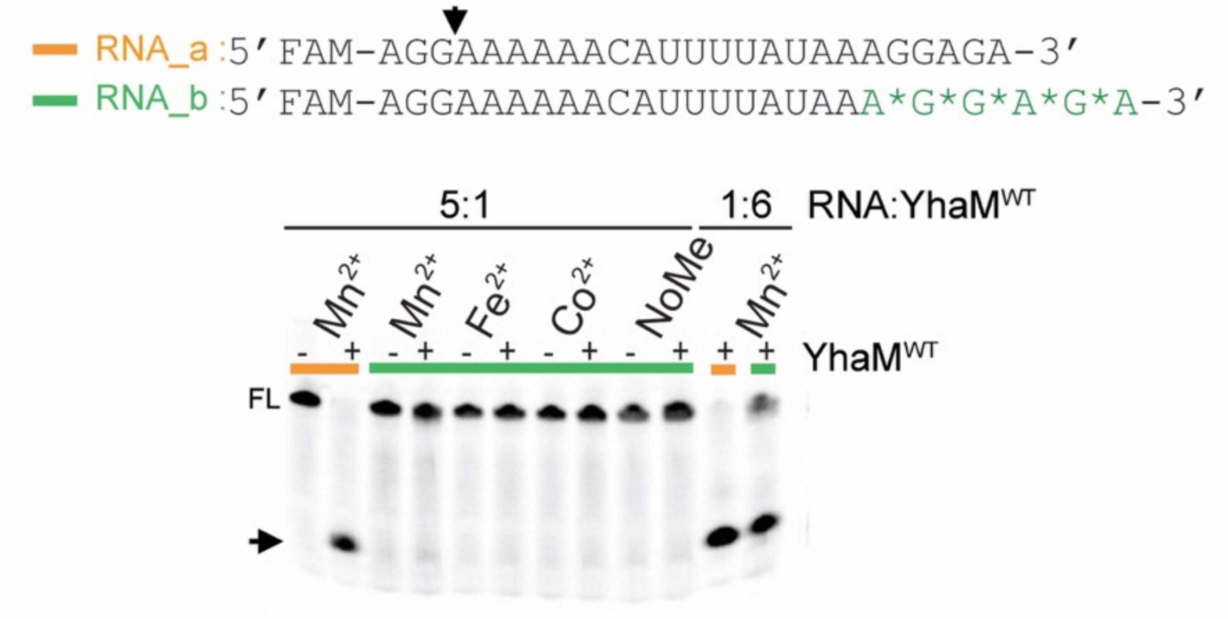

Figure S4. The 3’-5’ exonucleolytic activity of YhaM can be blocked by phosphonothioate bonds (Pt, green asterisk). *S. aureus* YhaM can efficiently cleave RNA_a with RNA-to-YhaM molar ratio of 5:1 but cannot cleave the Pt linkage-containing RNA_b with the identical sequence. At high protein concentrations (RNA:YhaM=1:6), YhaM can overcome 5 consecutive Pt bonds, leading to partial degradation of RNA_b. Both RNA_a and RNA_b were fluorescently labeled at their 5’-termini. FL, full length RNA; solid arrow, degraded product. NoMe, without metal cation. Samples were analyzed on a 15% TBE/urea polyacrylamide gel and the fluorescence signals were visualized on an iBright™ FL1500 imager.

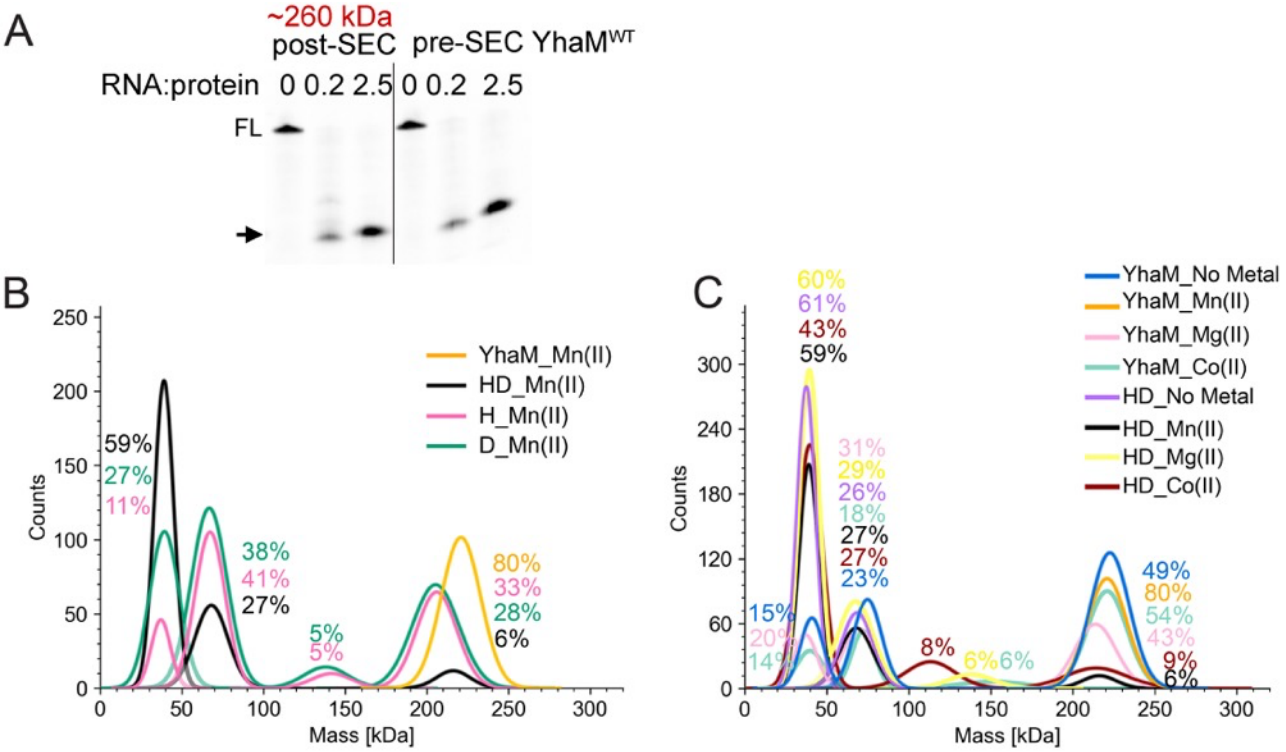

Figure S5. **Analysis of YhaM oligomerization. (A)** The hexameric YhaM^{WT} eluted from the size-exclusion chromatography (SEC, **Figure 7A**) is as active as the affinity column purified YhaM. *In vitro* RNA degradation assay was performed using RNA_a substrate in the presence of Mn(II). FL, full-length RNA_a; solid arrow, degraded fragment. Samples were analyzed on a 15% TBE/urea polyacrylamide gel and the fluorescence signals were visualized on an iBright^{TM} FL1500 imager. **(B)** The HD domain of YhaM is critical for multimerization. Distribution of YhaM oligomers in Mn(II) containing buffer as measured by the single molecule mass photometry.**(C)** HD domain mutants are severely impaired in oligomerization. Mn(II) promotes the greatest formation of oligomers whereas Mg(II) has an opposite effect. H, YhaM^{H192A}; D, YhaM^{D193A}; HD, YhaM$^{H192A/D193A}$.

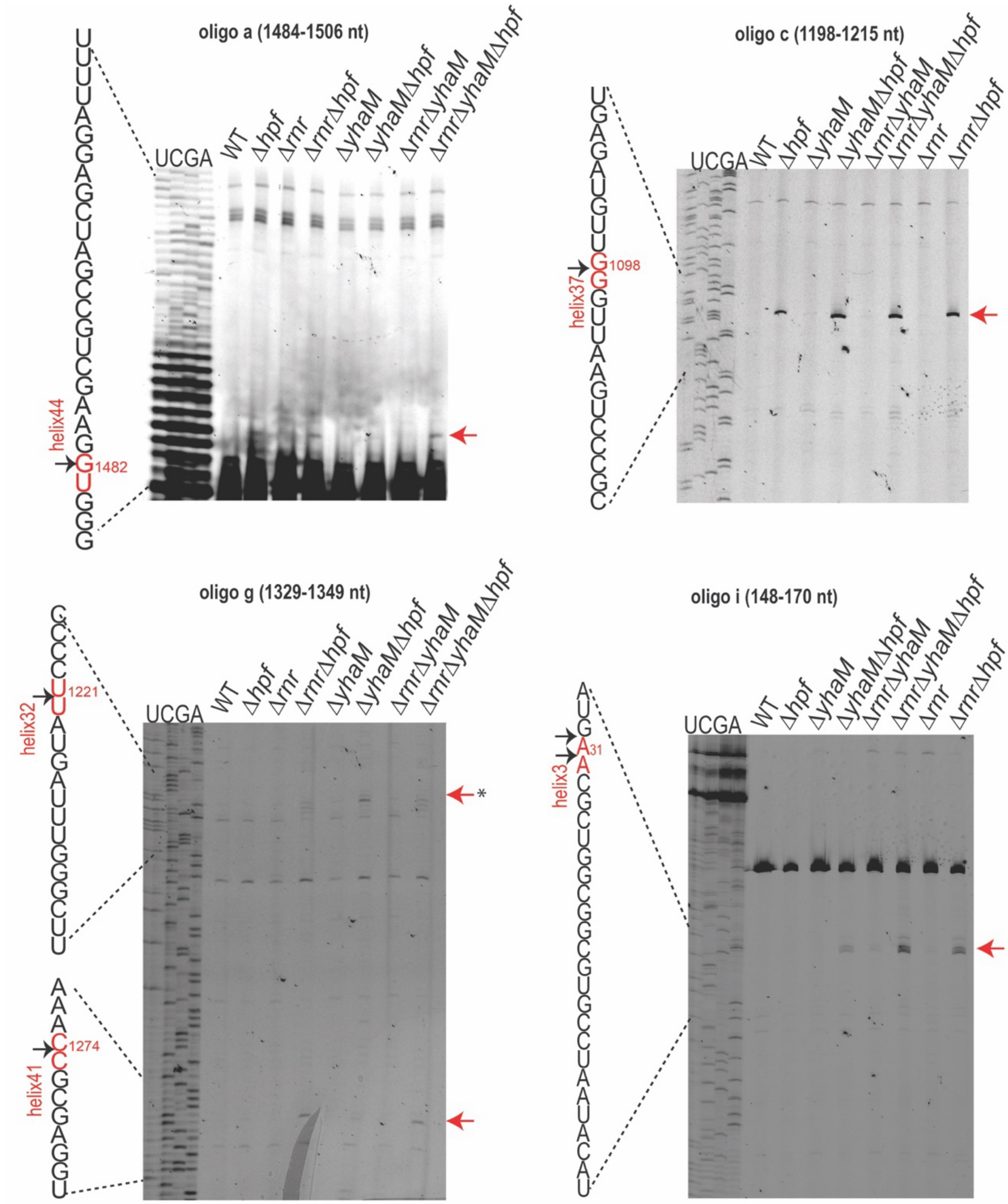

Figure S6. Primer extension mapping of the Hpf-protected 16S rRNA regions. Primer extension was performed using the 5'-end fluorescently labeled anti-sense oligos a, c, g or i (**Table S3**) to detect differentially cleaved rRNA helices h3, h32, h37, h41 and h44. Reverse transcriptase halts at 5'-end of a cleaved RNA template and generates a truncated cDNA that is subsequently analyzed on a 6% or 10% TBE-urea polyacrylamide gel. The red arrow indicates the position of a cleavage product. An asterisk indicates a fragment present only in the strain with both deletion of *yhaM* and *hpf* (Δ*yhaM*Δ*hpf* and Δ*yhaM*Δ*rnr*Δ*hpf*). 'UCGA' marks the sequencing ladder. Fluorescence signals were recorded on an iBright^{TM} FL1500 imager. The exact cleavage sites on the *S. aureus* 16S rRNA secondary structure are also illustrated in **Figure S7**.

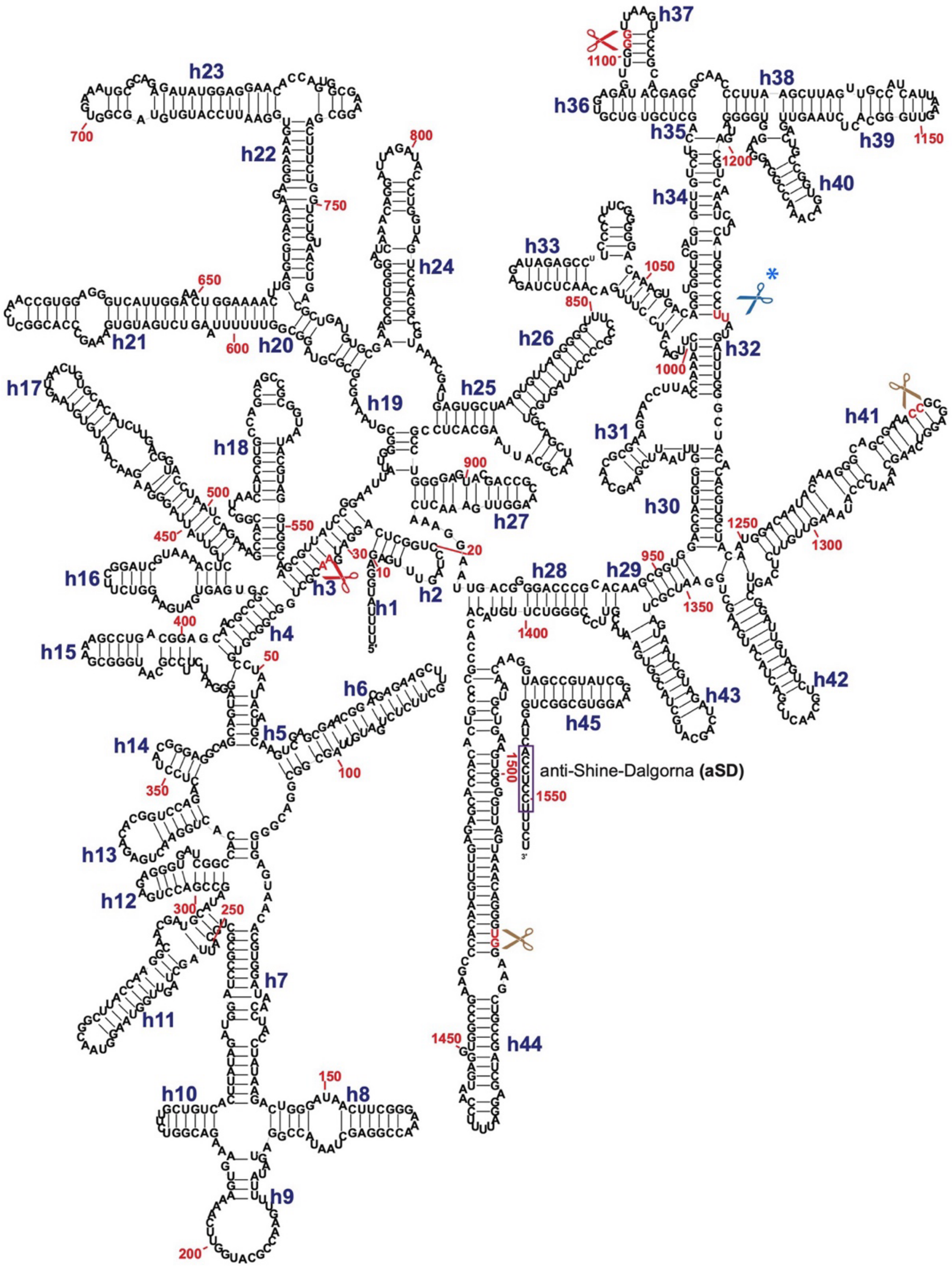

Figure S7. Secondary structure of the 16S rRNA showing the five RNase susceptible regions (h3, h32, h37, h41, and h44) in the absence of Hpf. A diagram of 16S rRNA from *S. aureus* USA300_FPR3757 (GenBank CP000255). The secondary structure is adapted from RNA central database (https://rnacentral.org/rna/URS00001B1A25/451515)(26). The scissors indicate the cleavage sites detected in the ∆*hpf* strains. A blue scissor with and asterisk marks the cleavage site unique to ∆*yhaM* and the brown scissors show the cleavage sites specific to ∆*rnr*∆*hpf* (see **Figure S6**)

SUPPLEMENTARY REFERENCES

1. Yoshida, H. and Wada, A. (2014) The 100S ribosome: ribosomal hibernation induced by stress. *Wiley Interdiscip Rev RNA*, **5**, 723-732.
2. Davis, A.R., Gohara, D.W. and Yap, M.N. (2014) Sequence selectivity of macrolide-induced translational attenuation. *Proc Natl Acad Sci U S A*, **111**, 15379-15384.
3. Ueta, M., Wada, C., Daifuku, T., Sako, Y., Bessho, Y., Kitamura, A., Ohniwa, R.L., Morikawa, K., Yoshida, H., Kato, T. *et al.* (2013) Conservation of two distinct types of 100S ribosome in bacteria. *Genes Cells*, **18**, 554-574.
4. Ueta, M., Wada, C. and Wada, A. (2010) Formation of 100S ribosomes in *Staphylococcus aureus* by the hibernation promoting factor homolog SaHPF. *Genes Cells*, **15**, 43-58.
5. Beckert, B., Turk, M., Czech, A., Berninghausen, O., Beckmann, R., Ignatova, Z., Plitzko, J.M. and Wilson, D.N. (2018) Structure of a hibernating 100S ribosome reveals an inactive conformation of the ribosomal protein S1. *Nature microbiology*, **3**, 1115–1121.
6. Khusainov, I., Vicens, Q., Bochler, A., Grosse, F., Myasnikov, A., Ménétret, J.F., Chicher, J., Marzi, S., Romby, P., Yusupova, G. *et al.* (2016) Structure of the 70S ribosome from human pathogen *Staphylococcus aureus*. *Nucleic Acids Res*, **44**, 10491–10504.
7. Matzov, D., Aibara, S., Basu, A., Zimmerman, E., Bashan, A., Yap, M.N.F., Amunts, A. and Yonath, A. (2017) The cryo-EM structure of hibernating 100S ribosome dimer from pathogenic *Staphylococcus aureus*. *Nat Commun*, **8**, 723.
8. Matzov, D., Bashan, A., Yap, M.F. and Yonath, A. (2019) Stress response as implemented by hibernating ribosomes: a structural overview. *FEBS J*, **286**, 3558-3565.
9. Polikanov, Y.S., Blaha, G.M. and Steitz, T.A. (2012) How hibernation factors RMF, HPF, and YfiA turn off protein synthesis. *Science*, **336**, 915-918.
10. Ueta, M., Yoshida, H., Wada, C., Baba, T., Mori, H. and Wada, A. (2005) Ribosome binding proteins YhbH and YfiA have opposite functions during 100S formation in the stationary phase of *Escherichia coli*. *Genes Cells*, **10**, 1103-1112.
11. Wada, A., Yamazaki, Y., Fujita, N. and Ishihama, A. (1990) Structure and probable genetic location of a "ribosome modulation factor" associated with 100S ribosomes in stationary-phase *Escherichia coli* cells. *Proc Natl Acad Sci U S A*, **87**, 2657-2661.
12. Vila-Sanjurjo, A., Schuwirth, B.S., Hau, C.W. and Cate, J.H. (2004) Structural basis for the control of translation initiation during stress. *Nat Struct Mol Biol*, **11**, 1054-1059.
13. Prossliner, T., Sørensen, M.A. and Winther, K.S. (2021) Hibernation factors directly block ribonucleases from entering the ribosome in response to starvation. *Nucleic Acids Res*, **49**, 2226-2239.
14. Liponska, A. and Yap, M.-N.F. (2021) Hibernation-promoting factor sequesters *Staphylococcus aureus* ribosomes to antagonize RNase R-mediated nucleolytic degradation. *mBio*, **2(4)**, e00334-00321.
15. Bechhofer, D.H. and Deutscher, M.P. (2019) Bacterial ribonucleases and their roles in RNA metabolism. *Crit Rev Biochem Mol Biol*, **54**, 242-300.
16. Condon, C., Pellegrini, O., Gilet, L., Durand, S. and Braun, F. (2021) Walking from *E. coli* to *B. subtilis*, one ribonuclease at a time. *C R Biol*, **344**, 357-371.
17. Durand, S. and Condon, C. (2021) RNases and Helicases in Gram-Positive Bacteria. *Microbiol Spectr*, **6**, 10.1128/microbiolspec.RWR-0003-2017.
18. Ingle, S., Chhabra, S., Chen, J., Lazarus, M.B., Luo, X. and Bechhofer, D.H. (2022) Discovery and initial characterization of YloC, a novel endoribonuclease in *Bacillus subtilis*. *RNA*, **28**, 227-238.
19. Fey, P.D., Endres, J.L., Yajjala, V.K., Widhelm, T.J., Boissy, R.J., Bose, J.L. and Bayles, K.W. (2013) A genetic resource for rapid and comprehensive phenotype screening of nonessential *Staphylococcus aureus* genes. *mBio*, **4**, e00537-00512.
20. Monk, I.R., Shah, I.M., Xu, M., Tan, M.W. and Foster, T.J. (2012) Transforming the untransformable: application of direct transformation to manipulate genetically *Staphylococcus aureus* and *Staphylococcus epidermidis*. *mBio*, **3**, e00277-00211.

21. Lee, C.Y., Buranen, S.L. and Ye, Z.H. (1991) Construction of single-copy integration vectors for *Staphylococcus aureus*. *Gene*, **103**, 101-105.
22. Stols, L., Gu, M., Dieckman, L., Raffen, R., Collart, F.R. and Donnelly, M.I. (2002) A new vector for high-throughput, ligation-independent cloning encoding a tobacco etch virus protease cleavage site. *Protein Expr Purif*, **25**, 8-15.
23. Basu, A. and Yap, M.N. (2016) Ribosome hibernation factor promotes Staphylococcal survival and differentially represses translation. *Nucleic Acids Res*, **44**, 4881-4893.
24. Maass, S., Sievers, S., Zühlke, D., Kuzinski, J., Sappa, P.K., Muntel, J., Hessling, B., Bernhardt, J., Sietmann, R., Völker, U. *et al.* (2011) Efficient, global-scale quantification of absolute protein amounts by integration of targeted mass spectrometry and two-dimensional gel-based proteomics. *Anal Chem*, **83**, 2677-2684.
25. Fischer, H., Polikarpov, I. and Craievich, A.F. (2004) Average protein density is a molecular-weight-dependent function. *Protein Sci*, **13**, 2825-2828.
26. The, R.C. (2019) RNAcentral: a hub of information for non-coding RNA sequences. *Nucleic Acids Res*, **47**, D221-D229.
